# Selenoprotein T Protects Endothelial Cells against Lipopolysaccharide-Induced Activation and Apoptosis

**DOI:** 10.3390/antiox10091427

**Published:** 2021-09-07

**Authors:** Dennis Merk, Johannes Ptok, Philipp Jakobs, Florian von Ameln, Jan Greulich, Pia Kluge, Kathrin Semperowitsch, Olaf Eckermann, Heiner Schaal, Niloofar Ale-Agha, Joachim Altschmied, Judith Haendeler

**Affiliations:** 1 Environmentally-Induced Cardiovascular Degeneration, Clinical Chemistry and Laboratory Diagnostics, Medical Faculty, University Hospital and Heinrich-Heine University Düsseldorf, 40225 Düsseldorf, Germany; dennis.merk@hhu.de (D.M.); philipp.jakobs@hhu.de (P.J.); pia.kluge@uni-potsdam.de (P.K.); semperina@gmail.com (K.S.); olaf.eckermann@hhu.de (O.E.); 2 Institute for Virology, Medical Faculty, University Hospital and Heinrich-Heine University Düsseldorf, 40225 Düsseldorf, Germany; Johannes.ptok@hhu.de (J.P.); schaal@uni-duesseldorf.de (H.S.); 3 Environmentally-Induced Cardiovascular Degeneration, Clinical Chemistry and Laboratory Diagnostics, Medical Faculty, University Hospital and Heinrich-Heine University Düsseldorf, Germany and IUF-Leibniz Research Institute for Environmental Medicine, 40225 Düsseldorf, Germany; florian.ameln@hhu.de (F.v.A.); jan.greulich@hhu.de (J.G.)

**Keywords:** APEX1(1-20), Selenoprotein T, lipopolysaccharide, endothelial cell activation, apoptosis

## Abstract

Sepsis is an exaggerated immune response upon infection with lipopolysaccharide (LPS) as the main causative agent. LPS-induced activation and apoptosis of endothelial cells (EC) can lead to organ dysfunction and finally organ failure. We previously demonstrated that the first twenty amino acids of the Apurinic/Apyrimidinic Endodeoxyribonuclease 1 (APEX1) are sufficient to inhibit EC apoptosis. To identify genes whose regulation by LPS is affected by this N-terminal APEX1 peptide, EC were transduced with an expression vector for the APEX1 peptide or an empty control vector and treated with LPS. Following RNA deep sequencing, genes upregulated in LPS-treated EC expressing the APEX1 peptide were identified bioinformatically. Selected candidates were validated by semi-quantitative real time PCR, a promising one was Selenoprotein T (SELENOT). For functional analyses, an expression vector for SELENOT was generated. To study the effect of SELENOT expression on LPS-induced EC activation and apoptosis, the SELENOT vector was transfected in EC. Immunostaining showed that SELENOT was expressed and localized in the ER. EC transfected with the SELENOT plasmid showed no activation and reduced apoptosis induced by LPS. SELENOT as well as APEX1(1-20) can protect EC against activation and apoptosis and could provide new therapeutic approaches in the treatment of sepsis.

## 1. Introduction

Sepsis can best be described as an overwhelming inflammatory condition, in which the body responds to an infection in a hyperactive, dysregulated way, which in turn results in life-threatening organ dysfunction and eventually septic shock. According to an estimate of the World Health Organization (WHO), sepsis affects more than 48 million people every year, potentially leading to 11 million deaths [1]. The basis for the pathophysiological responses in the context of sepsis is multifactorial. Therefore, except for the introduction of vasopressor agents 40 years ago, no new therapeutic principle for the treatment of sepsis has been developed until today.

Lipopolysaccharide (LPS) is an outer membrane component of Gram-negative bacteria. Most bacterial LPS molecules are thermostable and generate a pro-inflammatory stimulus for the immune system in humans. LPS is a serologically reactive bacterial toxin, and 1 to 2 mg in the bloodstream can be lethal. LPS can enter the bloodstream through intestinal absorption of the LPS produced by gut bacteria. Moreover, gut lesions and diet rich in lipids boost the transport across membranes into the systemic circulation [2]. Therefore, at the cellular level, endothelial cells (EC) are directly affected by LPS, which triggers their activation and ultimately apoptosis, leading to vascular leakage. Thus, it is undisputable that the loss of endothelial cell integrity is a mainstay of septic shock [3]. Hence, therapies that could prevent endothelial cell leakage or even restore endothelial cell integrity would be of tremendous value for patients and would address medical needs. EC with LPS affect the endothelial transcriptome by regulating the levels of numerous transcripts, not only of protein coding RNAs, but also of non-coding RNAs such as microRNAs and long non-coding RNAs [4,5]. Having pointed this out, it is a mystery to us that we failed to find any RNA deep sequencing data on LPS-induced transcriptome changes in the endothelium in the established databases such as the Gene expression omnibus (GEO), the European nucleotide archive (ENA), Short Sequence Archive (SRA) or ArrayExpress. However, such an in-depth transcriptome profiling combined with pathway analyses could provide novel targets for the development of new therapeutic principles for the treatment of sepsis, especially for protecting the endothelium. Therefore, one aim of this study was to perform a deep sequencing analysis in LPS-treated primary EC.

Moreover, we have recently shown that the first 20 amino acids of the Apurinic/Apyrimidinic Endodeoxyribonuclease 1 (APEX1) are sufficient to inhibit H_2_O_2_-induced apoptosis [6]. As the underlying molecular mechanisms initiating apoptosis are independent of the trigger, we hypothesized that this N-terminal APEX1 peptide, APEX1(1-20), could also interfere with LPS-induced apoptosis. Therefore, we included cells expressing the APEX1(1-20) in our deep sequencing analysis to find potential therapeutic targets for sepsis, possibly regulated by this peptide.

## 2. Materials and Methods

### 2.1. Cultivation of Primary Human Endothelial Cells and HEK293

Primary human endothelial cells (EC) were obtained from LONZA (Cologne, Germany) and cultured as previously described [7]. In detail, EC were cultured in endothelial basal medium supplemented with 1 μg/mL hydrocortisone, 12 μg/mL bovine brain extract, 50 μg/mL gentamicin, 50 ng/mL amphotericin B, 10 ng/mL epidermal growth factor (LONZA, Cologne, Germany) and 10% fetal bovine serum until the third passage. After detachment with trypsin, cells were grown for at least 20 h. All experiments were performed with EC in passage 3. HEK293 were cultured in DMEM GlutaMAX™ supplemented with 10% heat-inactivated fetal bovine serum and 1% penicillin/streptomycin and then used for the production of lentiviruses.

### 2.2. Lentiviral Production and Transduction of EC

Generation of VSV-G pseudotyped lentiviral particles and transduction of EC were performed as previously described [8]. Lentiviral titers were determined with the QuickTiter^TM^ Lentivirus Titer kit (Lentivirus-Associated HIV p24) (Biocat, Heidelberg, Germany). EC were transduced with a multiplicity of infection of approximately 20. The day after transduction the cells were washed three times, the medium replaced, and cells cultivated for another day before they were treated with 150 ng/mL LPS for 18 h.

### 2.3. Isolation of Total Cellular RNA

Cells were lysed using TRIzol^®^ (Thermo Fisher Scientific, Dreieich, Germany) and RNA was isolated according to the manufacturer’s instructions. RNAs were subjected to a second purification step using the RNeasy^®^ Mini kit (Qiagen, Hilden, Germany). RNA concentrations were measured using a NanoDrop™ 2000c (Thermo Fisher Scientific, Dreieich, Germany), and RNA integrity and purity were determined by agarose gel electrophoresis.

### 2.4. RNA Sequencing and Bioinformatic Analysis

RNA sequencing data were obtained from quadruplicate total RNA samples. Total RNAs used for transcriptome analyses were quantified using the Qubit^TM^ RNA HS Assay kit (Thermo Fisher Scientific, Dreieich, Germany) and quality was determined by capillary electrophoresis using the FragmentAnalyzer and the Total RNA Standard Sensitivity Assay (Agilent Technologies, Santa Clara, CA, USA). All samples in this study showed highest RNA Quality Numbers (RQN 10.0). Library construction and sequencing were performed at the Genomics and Transcriptomics Laboratory at the Biological Medical Research Centre (BMFZ) of the Heinrich-Heine University Düsseldorf. Library preparation was performed according to the manufacturer’s protocol using the TruSeq Stranded mRNA Assay kit (Illumina, San Diego, CA, USA). Briefly, 500 ng total RNA was used for mRNA capturing, fragmentation, synthesis of cDNA, adapter ligation and library amplification. Bead purified libraries were normalized and finally sequenced on the HiSeq 3000 system (Illumina San Diego, CA, USA) with a read setup of 1 × 150 bp. The bcl2fastq2 (version 2.17.1.4) tool was used to convert the bcl files to fastq files as well for adapter trimming and demultiplexing. GC-content, base-calling quality, adapter content and read length were measured using the tool FASTQC by Andrews (http://www.bioinformatics.babraham.ac.uk/projects/fastqc/ accessed on 17 August 2021) and MultiQC [9]. Reads were then trimmed or discarded based on their base calling quality and adapter content with Trimmomatic version 0.36 [10]. Subsequently, with the help of the SortMeRNA algorithm version 2.1b [11], the extent of rRNA depletion was measured by mapping the reads to rRNA databases. For alignment and the following analyses, the human genomic reference sequence (GRCh38) and annotation data (release 101) were downloaded from Ensembl [12] and BioMart [13]. For splice site usage analysis, the reads were then aligned to the human reference genome using the two-pass mapping protocol of the STAR aligner (2.5.4b) [14]. With help of the SAMtools software package [15], uniquely mapped reads were selected for creation of a gap table, listing the coordinates of every gap found in the alignment of the reads and the number of overlapping reads. For DGE analysis with the R package DESeq2 version 1.18.1 [16], count matrices were generated using the software salmon version 0.9.1 [17]. Significantly enriched gene sets were calculated, using the R package GOseq [18]. Scripts used for this work are publicly available at https://github.com/caggtaagtat/SELENOT (accessed on 17 August 2021). FASTQ file preparation and alignment were accomplished using custom BASH shell scripts in the environment of the High Performing Cluster of the Heinrich-Heine University Düsseldorf.

### 2.5. cDNA Synthesis

Total cellular RNA was reverse transcribed using the QuantiTect Reverse Transcription kit (Qiagen, Hilden Germany) according to the manufacturer’s instructions.

### 2.6. Polymerase Chain Reaction (PCR)

Endpoint PCRs were performed with MyTaq™ HS DNA Polymerase (Biocat, Heidelberg, Germany) according to manufacturer’s recommendations in a Bio-Rad T100 Thermal Cycler (Bio-Rad, Feldkirchen Germany). Reaction products were resolved on standard agarose gels.

Relative transcript levels were determined by semi-quantitative real-time PCR using cDNA as a template and the primaQUANT 2x qPCR-SYBR-Green-MasterMix (Steinbrenner, Wiesenbach, Germany), the transcript for the ribosomal protein L32 (RPL32), served as a reference. The PCR reactions were performed in a Rotor-Gene Q instrument (Qiagen, Hilden, Germany). Relative expression was calculated by the ΔC_t_ method [19].

The sequences of all primer used for PCR are listed in Supplementary Table S1.

### 2.7. Plasmids

A lentiviral expression vector for the first twenty amino acids of APEX1 was constructed by transferring the coding sequence for APEX1(1-20) with a C-terminal myc-tag from the previously published expression vector [6] into a lentiviral transfer vector, in which the transgene is expressed under the transcriptional control of the cytomegalovirus immediate early promoter/enhancer [8]. To generate an expression vector for human SELENOT with an N-terminal FLAG-tag, the SELENOT coding sequence together with the first 179 bp of the 3′-untranslated region of the human SELENOT gene containing the selenocysteine insertion sequence were amplified from a human EC cDNA using Q5^®^ High-Fidelity DNA Polymerase (New England Biolabs, Frankfurt, Germany). This fragment was inserted into pFLAG-CMV-2 (Sigma-Aldrich, Deisenhofen, Germany) opened with Not I and Xba I using the Gibson Assembly^®^ Cloning kit (New England Biolabs, Frankfurt, Germany) according to the manufacturer’s protocol. The construct was verified by DNA sequencing. Cloning details and the complete plasmid sequence are available upon request.

### 2.8. Transient Transfection of EC

Transient transfections of EC with plasmid DNA were performed using Superfect (Qiagen, Hilden, Germany) as previously described [20,21]. In detail, EC were transfected on 6 cm culture dishes with 3 µg plasmid DNA and 22.5 µL Superfect, or in 6-well plates with 1.2 µg plasmid DNA and 12 µL Superfect per well.

### 2.9. Immunostaining of EC

EC were fixed and permeabilized as described previously [7]. Afterwards, cells were incubated with an anti-FLAG-tag antibody (1:100, DYKDDDDK Tag Antibody (clone 8H8L17), Abfinity™, Cat. No. 701629, Invitrogen, Darmstadt, Germany). As secondary antibody, a goat anti-rabbit highly cross-adsorbed antibody coupled to Alexa Fluor 594 (1:500, Cat. No. A-11012, Invitrogen, Darmstadt, Germany) was used. For ICAM1 staining, an Alexa Fluor 488-coupled primary antibody (1:50, ICAM1/CD54 (15.2), Cat. No. SC-107 AF488, Santa Cruz Biotechnology, Heidelberg, Germany) was used. The endoplasmic reticulum (ER) was stained with an anti-Calnexin (clone C5C9) Alexa Fluor 488-conjugate (1:25, Cat. No. 38552, Cell Signaling, Technology, Frankfurt, Germany). Nuclei were counterstained with 4′,6-diamidino-2-phenylindole (DAPI) (100 ng/mL, Sigma-Aldrich, Deisenhofen, Germany). Images were taken using Zeiss microscopes (Axio Observer D1 or Axio Imager M2, magnification 400-fold, oil).

### 2.10. Immunoblotting

Cells were detached from the culture surface with a rubber policeman, centrifuged at 800× *g*, resuspended in radioimmunoprecipitation assay (RIPA) buffer (50 mM Tris-HCl pH 8.0, 150 mM NaCl, 1% (*v*/*v*) IGEPAL^®^-CA630, 0.1% (*w*/*v*) SDS and 0.5% (*w*/*v*) Na-Deoxycholate) supplemented with 1/100 volume of a protease inhibitor cocktail (Bimake, Munich, Germany) and lysed for 30 min on ice. The lysates were centrifuged at 18,000× *g* and 4 °C for 15 min and the supernatant was transferred to a fresh tube. Lysate proteins were separated by sodium-dodecyl-sulfate polyacrylamide gel electrophoresis according to standard procedures and transferred onto polyvinylidene difluoride membranes. After blocking with 5% milk powder in TBS (200 mM Tris-HCl pH 8.0, 300 mM NaCl, 100 mM KCl) with 0.1% (*v*/*v*) Tween-20 for 1 h at room temperature, membranes were incubated with an antibody directed against Caspase 3 (1:300 for detection of cleaved Caspase 3, 1:500 for uncleaved Caspase 3, Cat. No. 9662, Cell Signaling Technology, Frankfurt, Germany) and an anti α-Tubulin antibody (clone (DM1A), 1:50,000, Cat. No. T9026, Sigma-Aldrich, Deisenhofen, Germany). Antibodies were incubated overnight at 4 °C. The following day, membranes were incubated with secondary antibodies coupled to horseradish peroxidase (ECL^TM^ Anti-Rabbit or Anti-Mouse IgG, Horseradish Peroxidase linked whole antibody (from sheep), 1:5000; Cat. Nos. NA934V and NA931V, GE healthcare, Solingen, Germany). Detection was performed using ECL substrate (GE healthcare, Solingen, Germany) and X-ray films. Semi-quantitative analyses were performed on scanned X-ray films using Fiji [22].

### 2.11. Statistics

The number of experiments (n) given in the figure legends represents independent biological replicates, the data shown are mean ± SEM. Normal distribution for all data sets was confirmed by a Shapiro–Wilk test; homogeneity of variances (from means) between groups was verified by Levene’s test. Multiple comparisons were performed using one-way ANOVA with post-hoc Tukey LSD test.

## 3. Results

### 3.1. APEX1(1-20) Induces Specific Transcriptome Changes in EC in Response to LPS

To identify APEX1(1-20)-mediated transcriptome differences in the response of EC to LPS we performed RNA deep sequencing. For this purpose, primary human EC were transduced with either a lentiviral vector leading to moderate expression of APEX1(1-20) or an empty vector, respectively. Cells were then treated with 150 ng/mL active LPS or detoxified LPS as control. RNA from these cells was used for RNA deep sequencing and analyzed for differential gene expression (DGE). To identify APEX1(1-20)-specific transcriptome changes in response to LPS, we analyzed which genes were specifically regulated by LPS in the APEX1(1-20) expressing cells, but not in the cells transduced with the empty vector.

PCA analysis revealed that all samples from the cells treated with detoxified LPS cluster together, no matter whether the cells expressed APEX1(1-20) or not. The same held true for the LPS-treated cells (Supplementary Figure S1), showing a clear effect of LPS on the cellular transcriptome.

DGE analysis revealed that the APEX1(1-20) transcript derived from the expression vector was only detectable in the cells transduced with this vector, but not in the cells transduced with the empty vector. More importantly, expression of APEX1(1-20) alone did not appear to affect the overall transcriptome as changes in the expression of only a very small number of genes were observed (Supplementary Table S2).

In addition to the DGE analysis, we performed a gene set enrichment analysis (GSEA) focusing on genes that were significantly regulated by LPS exclusively in either the cells transduced with the empty vector or the cells expressing APEX1(1-20). As expected, cells transduced with the empty vector showed a significant enrichment of upregulated genes belonging to gene ontology (GO) terms related to immune responses including the response to bacteria and tumor necrosis factor signaling (Supplementary Table S3). Interestingly, we did not observe these changes in the presence of APEX1(1-20), and, moreover, genes belonging to the GO term cellular response to tumor necrosis factor were significantly downregulated in LPS-treated cells expressing the APEX1 peptide (Supplementary Table S4). These data support the assumption that APEX1(1-20) might provide protection against endothelial cell activation and apoptosis via alteration of the transcriptional responses to LPS treatment.

**Figure 1:**
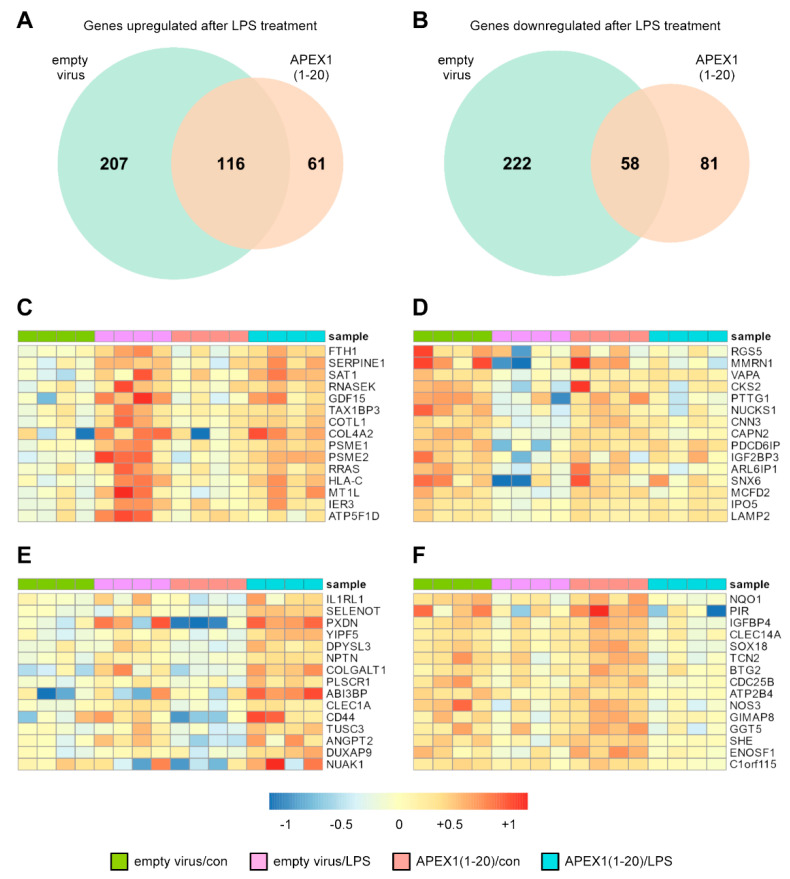
APEX1(1-20) induces specific transcriptome changes in EC in response to LPS. (**A**–**F**) EC were transduced with a lentiviral expression vector for APEX1(1-20) or an empty virus and treated with 150 ng/mL detoxified (con) or active LPS (LPS) for 18 h. RNAs from the transduced cells were subjected to RNA deep sequencing. Differential gene expression was calculated using the R package DESeq2. Wald test from DESeq2 was used to calculate the significance of the change in the expression. (**A**,**B**) Venn-diagrams for genes upregulated (**A**) or downregulated (**B**) after LPS treatment of empty virus transduced cells and cells expressing APEX1(1-20). (**C**–**F**) Heatmaps of genes significantly differentially expressed upon LPS treatment of cells transduced with the empty virus or cells expressing APEX1(1-20). Shown are the 15 top ranked genes from Supplementary Tables S7–S10. The color depicts the normalized expression relative to the respective mean in all samples. (**C**) Genes uniquely upregulated after LPS treatment of empty virus transduced cells. (**D**) Genes uniquely downregulated after LPS treatment of empty virus transduced cells. (**E**) Genes uniquely upregulated after LPS treatment of APEX1(1-20) expressing cells. (**F**) Genes uniquely downregulated after LPS treatment of APEX1(1-20) expressing cells.

In the DGE analysis, we found that after LPS treatment, 323 genes were significantly upregulated in cells transduced with the empty vector and 280 were downregulated. In contrast, in the cells expressing APEX1(1-20), only 177 genes were upregulated by LPS and 139 genes were downregulated (Figure 1A,B and Supplementary Tables S5 and S6). Thus, the expression of only roughly half as many genes appeared to be affected by the presence of APEX1(1-20).

Notably, we observed clearly different LPS responses in cells expressing APEX1(1-20) when compared to cells transduced with the empty vector (Figure 1C–F and Supplementary Tables S7–S10).

For functional studies, we focused on genes whose expression is upregulated by LPS only in cells expressing the small APEX1 peptide as the corresponding proteins might evoke APEX1(1-20)-dependent protective effects in EC, which could be of interest in a therapeutic setting.

### 3.2. Expression of PXDN and SELENOT Is Specifically Upregulated after LPS Treatment of EC Expressing APEX1(1-20)

As a prerequisite for functional studies, we first validated the regulation of the top-ranked candidates, which, according to the RNA sequencing data, should be expressed to levels allowing reliable detection and quantification.

IL1RL encodes an Interleukin 1 Receptor-like protein, which belongs to a family of ten distinct but structurally related receptors. These proteins serve either as ligand binding or accessory chains and some act as signaling inhibitors. Moreover, two members of this family are orphan receptors [23]. Therefore, IL1RL1 is part of a complex signaling network and one could easily envision that—due to this redundancy—interference with this network might be compensated or evoke unwanted side effects.

Peroxidasin (PXDN), originally described as Vascular Peroxidase 1, is a heme-containing peroxidase, which shows highest expression in the heart and the vascular wall [24]. The protein is rapidly secreted [25] and required for formation of the vascular basement membrane by reinforcing fibrillar network assembly in the extracellular matrix through formation of sulfilimine bonds [26]. It has recently been shown that PXDN promotes angiogenesis [27] and, furthermore, is essential for endothelial cell survival [28].

Selenoprotein T (SELENOT) is a member of the selenoprotein family, whose members are characterized by containing one or more selenocysteine residues, frequently in enzymatically active sites [29]. SELENOT is the most highly conserved selenoprotein throughout evolution [30], suggestive of an essential function, which is underscored by the early embryonic lethality of mice in which the *selenot* gene is constitutively disrupted [31]. SELENOT is one of 7 out of 25 human selenoproteins localized to the ER [32]. The expression of SELENOT, like all other selenoproteins, depends on dietary selenium as shown by a reduced expression in chicken stomach after 55 days on a selenium-deficient diet. Moreover, this regimen resulted in stress injuries [33]. In addition, SELENOT protects kidney cells against cisplatin-induced apoptosis [34]. These observations go along with the notion that ER-resident selenoproteins are critical in cellular stress responses [35].

**Figure 2:**
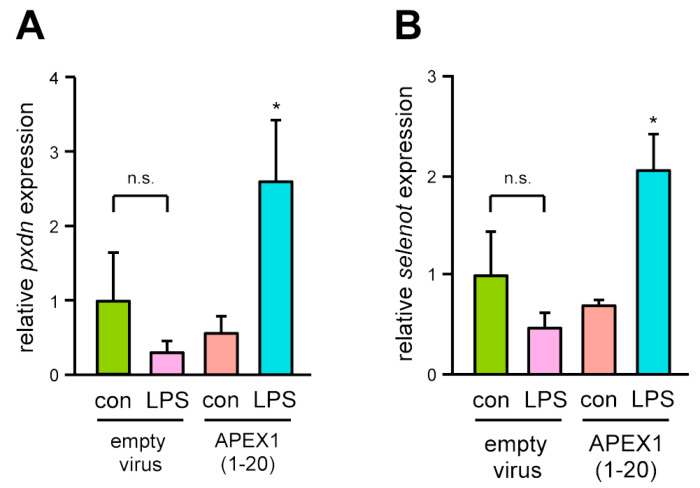
LPS induces upregulation of PXDN and SELENOT expression specifically in EC expressing APEX1(1-20). Transcript levels of PXDN (**A**) and SELENOT (**B**) in EC transduced with an empty virus or the expression vector for APEX1(1-20) and treated with 150 ng/mL detoxified (con) or active LPS (LPS) for 18 h were analyzed by semi-quantitative real-time PCR; RPL32 served as reference (data are mean ± SEM, *n* = 4, * *p* < 0.05 vs. APEX1(1-20)/con, n.s. = not significant, one-way ANOVA with post-hoc Tukey LSD test).

For the reasons explained above, we did not follow up on IL1RL, but validated the regulation of PXDN and SELENOT by semi-quantitative real-time PCR (Figure 2).

The real-time PCR analysis corroborated the deep sequencing data as for both genes, an upregulation of the transcript level by LPS was only observed in the cells expressing APEX1(1-20). Although PXDN has already been characterized with respect to protective functions in EC [28], these data provide independent proof for the validity of the experimental approach. The second protein, SELENOT, for which no functions in endothelial activation and apoptosis have been described so far, was chosen for functional analyses.

### 3.3. Generation of a SELENOT Expression Vector and Intracellular Localization of the Overexpressed Protein

To study the impact of SELENOT on endothelial cell functions affected by LPS, we generated an expression vector, which contained a FLAG-epitope tag allowing the identification of the overexpressed protein. For the generation of this expression vector, an aspect unique to selenoproteins had to be taken into account. Selenocysteine (Sec) residues in selenoproteins are not the product of a post-translational modification, but are rather incorporated already during translation by using one of the translation termination codons, namely UGA, for binding of the selenocysteine tRNA (tRNA^Sec^) to the mRNA. This translational recoding of the UGA codon involves a so-called selenocysteine insertion sequence (SECIS) in the 3′-untranslated region (UTR) of the transcript. The SECIS, which is not highly conserved on the sequence level, forms a stem-loop structure that is required for recruitment of the tRNA^Sec^ to the UGA codon [36]. Consequently, the lack of a SECIS leads to premature translation termination, when the ribosome encounters the first UGA within the open reading frame. Therefore, we included—besides the SELENOT open reading frame—a portion of the SELENOT 3′-UTR including the SECIS in the expression vector.

**Figure 3:**
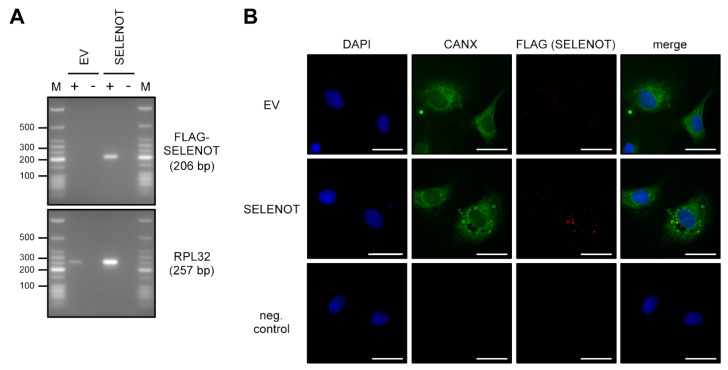
Overexpressed SELENOT is localized in the ER. (**A**,**B**) EC were transfected with the FLAG-SELENOT expression vector (SELENOT) or an empty vector (EV). Expression and localization of exogenously expressed SELENOT was verified on the RNA (**A**) and protein (**B**) level. (**A**) Expression of SELENOT was analyzed by reverse transcription polymerase chain reaction (RT-PCR). Therefore, RNA was isolated from the transfected cells and cDNA was synthesized in the presence (+) or absence (−) of reverse transcriptase. Amplification was performed with primers specifically detecting the FLAG-SELENOT fusion transcript, the housekeeping gene RPL32 served as control. Amplification products were resolved by agarose gel electrophoresis, the expected fragment sizes are specified, numbers on the left indicate selected DNA size markers (M). (**B**) Localization of FLAG-SELENOT was examined by immunostaining and fluorescence microscopy. Cells were stained with an antibody directed against Calnexin (CANX), a marker for the ER (green) and an anti-FLAG antibody (red). Nuclei were counterstained with DAPI (blue); merge is the overlay of all channels (scale bar = 30 μm).

We first analyzed the expression of FLAG-SELENOT after transient transfection of EC on the RNA level by reverse transcriptase PCR (Figure 3A). We then determined the intracellular localization of the overexpressed FLAG-SELENOT protein by immunofluorescence. As demonstrated by colocalization with the ER-resident protein Calnexin (Figure 3B), FLAG-SELENOT was localized in the ER.

### 3.4. SELENOT Overexpression Inhibits LPS-Induced Endothelial Cell Activation

**Figure 4:**
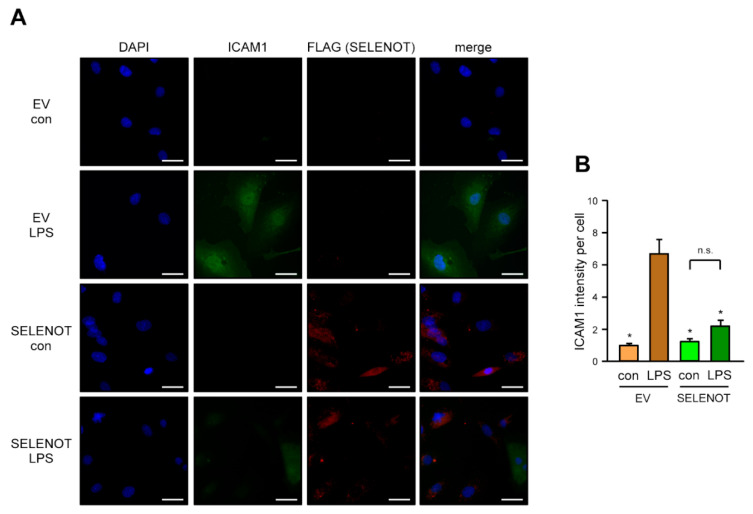
SELENOT suppresses LPS-induced upregulation of ICAM1. (**A**,**B**) EC were transfected with the FLAG-SELENOT expression vector (SELENOT) or an empty vector (EV) and treated with 150 ng/mL detoxified (con) or active LPS (LPS) for 18 h. FLAG-SELENOT and ICAM1 were detected by immunofluorescence. Cells were stained with an antibody directed ICAM1 (green) and an anti-FLAG antibody (red). Nuclei were counterstained with DAPI (blue); merge is the overlay of all channels. (**A**) Representative immunostaining (scale bar = 30 μm). (**B**) Quantitation of ICAM1 levels. The intensity of the green fluorescence per cell was measured using Fiji; in the cells transfected with the SELENOT expression vector, only FLAG-SELENOT positive cells were included (data are mean ± SEM, *n* = 4, * *p* < 0.05 vs. EV/LPS, n.s. = not significant, one-way ANOVA with post-hoc Tukey LSD test).

Having demonstrated that FLAG-SELENOT is localized in the ER, we next investigated the effect of SELENOT on LPS-induced endothelial cell activation. Therefore, FLAG-SELENOT was expressed in EC as before. After treatment with 150 ng/mL LPS for 18 h, ICAM1—a marker for endothelial cell activation—was detected. As expected, LPS upregulated ICAM1 protein levels in empty vector transfected EC. This upregulation was completely inhibited in cells, in which SELENOT is overexpressed (Figure 4).

### 3.5. SELENOT Overexpression Inhibits LPS-Induced Endothelial Cell Apoptosis

**Figure 5:**
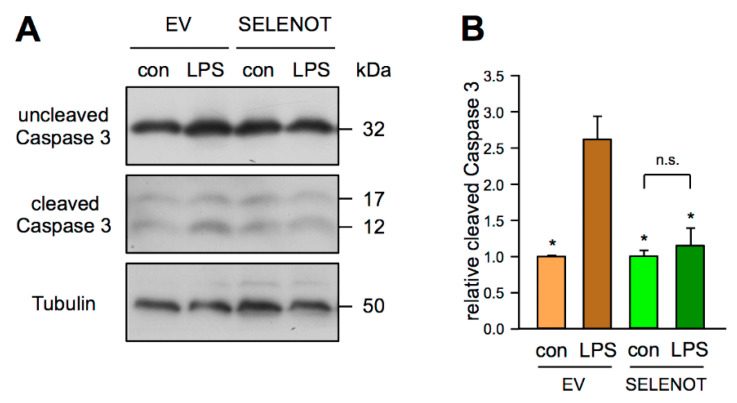
SELENOT suppresses apoptosis induction by LPS. (**A**,**B**) EC were transfected with the FLAG-SELENOT expression vector (SELENOT) or an empty vector (EV) and treated with 150 ng/mL detoxified (con) or active LPS (LPS) for 18 h. Uncleaved and cleaved Caspase 3 were detected by immunoblot, Tubulin served as loading control. (**A**) Representative immunoblot. (**B**) Semi-quantitative analysis of relative amounts of cleaved Caspase 3 (data are mean ± SEM, *n* = 4, * *p* < 0.05 vs. EV/LPS, n.s. = not significant, one-way ANOVA with post-hoc Tukey LSD test).

Besides endothelial cell activation, LPS also induces apoptosis of EC [37]. Therefore, we determined Caspase 3 cleavage as a marker for apoptosis in EC. As for ICAM1, LPS increased Caspase 3 cleavage in cells not expressing SELENOT. On the contrary, overexpression of SELENOT completely blunted apoptosis induction by LPS (Figure 5).

In conclusion, SELENOT, which is upregulated by LPS in EC expressing APEX1(1-20), seems to be an important mediator of the protective effects of APEX1(1-20) and could thus be of interest as an adjuvant therapeutic agent in endotoxemia.

## 4. Discussion

The major findings of the present study are the first RNA deep sequencing analysis of LPS-induced changes in primary human EC and the identification of a protective role of APEX1(1-20) and SELENOT in LPS-induced endothelial cell activation and apoptosis.

With respect to the possibility of using an APEX1(1-20) peptide or a related small molecule as a therapeutic agent, it has to be noted that APEX1(1-20) does not change the transcriptome when compared to empty virus transduced cells. Thus, there is no evidence of potential side effects induced by APEX1(1-20) in the endothelium. As expected, LPS treatment induced typical pathways known in sepsis. Those upregulated genes upon LPS treatment in cells not expressing APEX1(1-20) are found, for example, under the GO terms cellular response to tumor necrosis factor, tumor necrosis factor-mediated signaling pathway, and plasma membrane (Supplementary Table S3). It has been known for years that tumor necrosis factor induces endothelial cell activation [38] and apoptosis [39]. Therefore, activation of those pathways is a typical answer of the endothelium to LPS, which in turn leads to loss of endothelial integrity and barrier function. Loss of endothelial cell integrity is a mainstay of septic shock [3], because LPS can enter the systemic circulation destroy endothelial cell integrity, thereby leading to multiple organ failure. Thus, an additional therapy protecting the integrity of the endothelium would be of tremendous interest. Interestingly, APEX1(1-20) leads to reduced responses of the tumor necrosis factor pathways (Supplementary Table S4). Hence, APEX1(1-20) or its downstream targets could be of interest as potential therapeutic options. Therefore, we specifically focused on those targets induced by APEX1(1-20) in the presence of LPS in EC to identify potential candidates. Indeed, we found SELENOT to be upregulated upon APEX1(1-20).

SELENOT is an ER-resident selenoprotein, which is associated with the ER membrane and required to maintain ER redox homeostasis. It is needed to cope with intracellular stress conditions and is one of the most important selenoproteins [30].

As mentioned before, the expression of all selenoproteins depends on selenium. However, there seems to be a hierarchy in the sensitivity of different selenoproteins with respect to selenium levels and SELENOT seems to respond more avidly to selenium depletion than several other proteins of this family [40]. It has been estimated that up to one in seven people worldwide have a low dietary selenium intake [41] and it is clear that proper endothelial functionality depends on an adequate selenium supply [42]. Even more interesting is the observation that selenium serum levels are dramatically reduced in critically ill patients with sepsis [43]. Therefore, selenium supplementation seems to be an obvious supplementary treatment option for sepsis and possibly the protection of the endothelium in this disease. In this context, it is interesting to note that selenium pretreatment or supplementation alleviates some of the deleterious effects of LPS. In the murine macrophage cell line RAW264.7, LPS induced immunological stress as shown by the upregulation of multiple inflammation-related genes. This was accompanied by a reduction in the relative *selenot* mRNA level. Pretreatment with selenium partially rescued this downregulation and had only a very modest effect on the expression of the inflammation-related genes [44]. In mice, LPS-induced myocardial dysfunction, oxidative stress and apoptosis in the heart could be attenuated when the animals were put on a selenium-supplemented diet 2 weeks prior to LPS treatment [45]. Again, this pretreatment did not completely restore heart functionality or prevent oxidative stress and apoptosis induction evoked by LPS. Our experiments did not show a significant downregulation of *selenot* expression in LPS-treated EC, although there seems to be a trend in this direction. On the contrary, the cells expressing APEX1(1-20) showed an upregulation of *selenot* RNA levels of approximately threefold after LPS treatment. This clearly indicates that the small APEX1 peptide can convey a protective outcome, which is much stronger than the effects observed with selenium supplementation or pretreatment.

Up to now, the precise molecular functions of SELENOT have not been elucidated. Nevertheless, a peptide derived from SELENOT has already been used in animal models. Rocca et al. demonstrated that this SELENOT-derived peptide—including the active catalytic site corresponding to the sequence FQICVSUGYR—applied after ischemia and prior to reperfusion is able to protect the heart from ischemia/reperfusion injury. This protection was attributed to a reduction in oxidative stress and inhibition of apoptosis [46]. This is in accordance with our study presented here, in which we demonstrate that SELENOT completely inhibited LPS-induced activation and apoptosis in human primary EC.

The same peptide was applied in a cell-permeable form in a mouse model for Parkinson’s disease, where it protected dopaminergic neurons. This effect was also associated with reduced oxidative stress and Caspase 3 activity [47].

Based on the protective effects of this SELENOT peptide in such different organs as the brain and the heart, it is conceivable that it could exert its protective functions also in the vasculature in the setting of sepsis.

Given the high numbers of patients and the up to 11 million deaths per year due to sepsis, a protection of the endothelium as an additional additive therapy could be of tremendous importance. The metabolic response to sepsis entails the rapid breakdown of intracellular reserves of proteins, carbohydrates and fat. This is accompanied by an increase in ER stress. An increase in SELENOT or application of a peptide could dampen this stress and maintain the ER homeostasis, counteracting the overshooting responses of the body to sepsis.

## 5. Conclusions

In conclusion, our data presented here suggest that APEX1(1-20) and SELENOT are promising therapeutic options for the treatment of sepsis to protect the endothelium and thus, to prevent endothelial cell leakage or even to restore endothelial cell integrity. This would be of tremendous value for patients and would potentially lower the numbers of septic shock, multiple organ failure and deaths.

## Data Availability

Data are contained within the article. The RNA sequencing data used to support the findings of this study have been deposited at ArrayExpress under accession number E-MTAB-10936 (https://www.ebi.ac.uk/arrayexpress/09/2021, accessed on 17 August 2021).

## Supplementary Materials

The following are available online at https://www.mdpi.com/article/10.3390/antiox10091427/s1, Figure S1: Principal component analysis, Table S1: Primer pairs used for endpoint PCR and semi-quantitative real-time PCR. Primer pairs used for endpoint PCR and semi-quantitative real-time PCR, Table S2: Differential gene expression analysis for genes regulated byexpression of APEX1(1-20), Table S3: Overrepresented GO terms in genes upregulated by LPS exclusively in cells not expressing APEX1(1-20), Table S4: Overrepresented GO terms in genes downregulated by LPS exclusively in cells expressing APEX1(1-20), Table S5: Differentially expressed genes upon LPS treatment of cells not expressing APEX1(1-20), Table S6: Differentially expressed genes upon LPS treatment of cells expressing APEX1(1-20), Table S7: Genes upregulated by LPS exclusively in cells that do not express APEX1(1-20), Table S8: Genes downregulated by LPS exclusively in cells that do not express APEX1(1-20), Table S9: Genes upregulated by LPS exclusively in cells that express APEX1(1-20), Table S10: Genes downregulated by LPS exclusively in cells that express APEX1(1-20).
