# Supplementary material for: Selenoprotein T Protects Endothelial Cells against Lipopolysaccharide-Induced Activation and Apoptosis
Source: Antioxidants (Basel). 2021 Sep 7;10(9):1427. doi: 10.3390/antiox10091427 (PMC8469382; doi:10.3390/antiox10091427)
Supplement: Supplementary file 1 [file antioxidants-10-01427-s001.zip › antioxidants-1364794-SI.pdf]

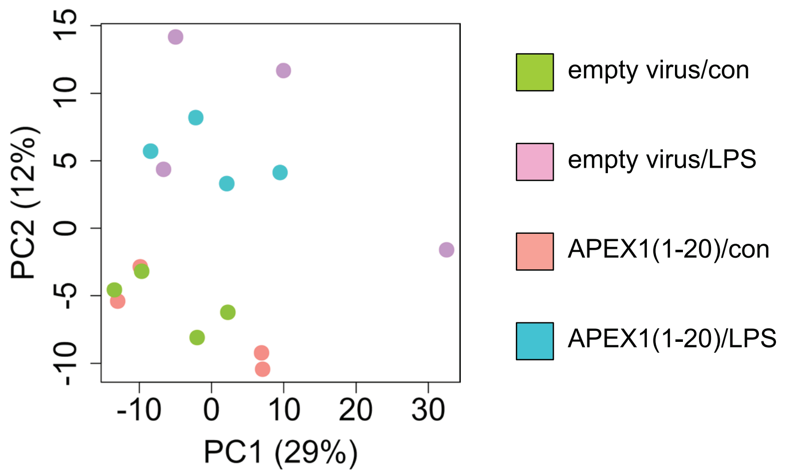

Supplementary figure S1: Principal component analysis. Endothelial cells were transduced with a lentiviral expression vector for APEX1(1-20) or an empty virus and treated with detoxified (con) or active LPS (LPS). and subjected to RNA deep sequencing.

Supplementary table S1: Primer pairs used for endpoint PCR and semi-quantitative real-time PCR.
Shown are the sequences of the primers in 5'→3' direction and the expected amplification products.

transcript	primer		sequence		amplification product
RPL32	hmRPL32 Ex02 for1	5'-	GTGAAGCCCAAGATCGTCAA	-3'	257 bp
	hmRPL32 Ex03 rev1	5'-	TTGTTGCACATCAGCAGCAC	-3'	
PXDN	hPXDN Ex20/21 for1	5'-	CGGAAAATACCCAGTGTTGGGAG	-3'	223 bp
	hPXDN Ex22 rev1	5'-	TGGTGTTGTTGGCGTGAGATTC	-3'	
SELENOT	hSELENOT Ex01 for	5'-	GCGTGCCCAGCAAGAGATTA	-3'	99bp
	hSELENOT Ex02 rev1	5'-	ACTCCTCAAACACCCGCCTA	-3'	
FLAG-SELENOT	FLAG for2	5'-	CAAAGACGATGACGACAAGC	-3'	206 bp
	hSELENOT Ex02 rev1	5'-	ACTCCTCAAACACCCGCCTA	-3'	

Supplementary Table S2: Differential gene expression analysis for genes regulated by expression of APEX1(1-20). DGE was calculated using the R package DESeq2 in samples of cells transduced with the lentivirus expressing APEX1(1-20) versus cells transduced with the empty virus both treated with detoxified LPS. The L2FC (Log 2-fold change) states the ave-rage difference in gene expression between the two cell populations. Positive L2FC values denote upregulation by APEX1(1-20) expression, negative values downregulation. Wald test from DESeq2 was used to calculate the significance of the change in the expression. The adjusted p-values take the number of tested genes into account, the threshold for the adjusted p-value was set to 0.05. APEX1(1-20)-myc represents the transcript originating from the APEX1(1-20) expression vector, which codes for a fusion between the APEX1 peptide and a myc epitope tag.

gene name	Ensembl gene ID	L2FC	p-value	adusted p-value
APEX1(1-20)-myc	NA	9,861	3,73E-40	2,92E-36
LRTOMT	ENSG00000184154	0,653	5,85E-11	3,06E-07
SRP9P1	ENSG00000180581	-26,509	5,11E-10	2,00E-06
AL358472.7	NA	21,631	2,86E-08	8,99E-05
CR354443.1	NA	-23,191	6,74E-08	1,51E-04
H3P6	ENSG00000235655	-22,915	6,69E-08	1,51E-04
KCNJ15	ENSG00000157551	-0,848	1,05E-06	2,06E-03
CAMSAP3	ENSG00000076826	1,139	7,29E-06	1,21E-02
GJA5	ENSG00000265107	0,484	7,73E-06	1,21E-02
GDF7	ENSG00000143869	0,397	9,76E-06	1,39E-02
CCL2	ENSG00000108691	0,324	1,80E-05	2,35E-02
HNRNPCP1	ENSG00000258900	17,502	4,12E-05	4,97E-02

Supplementary Table S3: Overrepresented GO terms in genes upregulated by LPS exclusively in cells not expressing APEX1(1-20). Gene set enrichment analysis (GSEA) was applied to genes from the DGE analysis, which were significantly upregulated by LPS exclusively in cells transduced with the empty virus GSEA was performed using R package goseq with a treshold on the adjusted p-values of 0.05. numDEInCat: number of differentially expressed genes belonging to the respective GO term; numInCat: number of genes related to the GO term.

GO ID	GO term	overrepresented p-value	adjusted p-value	numDEInCat	numInCat
GO:0005886	plasma membrane	8,50E-08	8,19E-05	101	2946
GO:0002479	antigen processing and presentation of exogenous peptide antigen via MHC class I, TAP-dependent	5,13E-06	2,95E-03	10	65
GO:0050852	T cell receptor signaling pathway	8,51E-06	4,63E-03	13	134
GO:0033209	tumor necrosis factor-mediated signaling pathway	9,82E-06	5,18E-03	12	102
GO:0019722	calcium-mediated signaling	1,05E-05	5,38E-03	8	49
GO:0002250	adaptive immune response	1,11E-05	5,45E-03	11	92
GO:0006959	humoral immune response	1,13E-05	5,45E-03	6	22
GO:0045766	positive regulation of angiogenesis	1,25E-05	5,86E-03	11	100
GO:0019955	cytokine binding	2,55E-05	1,16E-02	6	27
GO:0002486	antigen processing and presentation of endogenous peptide antigen via MHC class I via ER pathway, TAP-independent	3,90E-05	1,61E-02	3	3
GO:0046977	TAP binding	3,90E-05	1,61E-02	3	3
GO:0009395	phospholipid catabolic process	4,21E-05	1,69E-02	4	11
GO:0005604	basement membrane	4,36E-05	1,71E-02	8	71
GO:0016020	membrane	5,34E-05	2,00E-02	159	5600
GO:0016032	viral process	6,23E-05	2,29E-02	26	520
GO:0071356	cellular response to tumor necrosis factor	8,18E-05	2,87E-02	10	92
GO:1990111	spermatoproteasome complex	8,70E-05	2,99E-02	3	4
GO:0035455	response to interferon-alpha	9,63E-05	3,25E-02	4	10
GO:0010466	negative regulation of peptidase activity	1,07E-04	3,48E-02	7	46
GO:0030414	peptidase inhibitor activity	1,11E-04	3,49E-02	7	46
GO:0009617	response to bacterium	1,15E-04	3,49E-02	8	66
GO:0010951	negative regulation of endopeptidase activity	1,17E-04	3,49E-02	8	64
GO:0042270	protection from natural killer cell mediated cytotoxicity	1,18E-04	3,49E-02	3	4
GO:0001968	fibronectin binding	1,18E-04	3,49E-02	5	22
GO:0004867	serine-type endopeptidase inhibitor activity	1,40E-04	4,06E-02	6	37
GO:0019882	antigen processing and presentation	1,45E-04	4,15E-02	6	32

Supplementary Table S4: Overrepresented GO terms in genes downregulated by LPS exclusively in cells expressing APEX1(1-20). Gene set enrichment analysis (GSEA) was applied to genes from the DGE analysis, which were significantly downregulated by LPS exclusively in cells transduced with the lentivrus expressing APEX1(1-20). GSEA was performed using R package goseq with a treshold on the adjusted p-values of 0.05. numDEInCat: number of differentially expressed genes belonging to the respective GO term; numInCat: number of genes related to the GO term.

GO ID	GO term	overrepresented p-value	adjusted p-value	numDEInCat	numInCat
GO:0008217	regulation of blood pressure	4,11E-08	4,89E-04	7	38
GO:0005615	extracellular space	7,26E-08	4,89E-04	26	853
GO:0032355	response to estradiol	8,53E-08	4,89E-04	9	87
GO:0005576	extracellular region	2,65E-07	1,14E-03	32	1306
GO:0031994	insulin-like growth factor I binding	2,85E-06	9,81E-03	4	12
GO:0005887	integral component of plasma membrane	5,11E-06	1,46E-02	22	839
GO:0016324	apical plasma membrane	6,85E-06	1,56E-02	11	236
GO:0009612	response to mechanical stimulus	8,14E-06	1,56E-02	6	53
GO:0048247	lymphocyte chemotaxis	8,17E-06	1,56E-02	4	13
GO:0009986	cell surface	9,27E-06	1,59E-02	15	444
GO:0031526	brush border membrane	1,58E-05	2,46E-02	5	36
GO:0005113	patched binding	2,55E-05	3,37E-02	3	7
GO:0071356	cellular response to tumor necrosis factor	2,55E-05	3,37E-02	7	93
GO:0006874	cellular calcium ion homeostasis	3,35E-05	4,12E-02	6	69
GO:0002548	monocyte chemotaxis	4,05E-05	4,59E-02	4	20
GO:0031995	insulin-like growth factor II binding	4,27E-05	4,59E-02	3	8

Supplementary Table S5: Differentially expressed genes upon LPS treatment of cells not expressing APEX1(1-20). DGE calculated using the R package DESeq2 comparing samples of cells transduced with an empty virus and treated with active LPS versus treated with detoxified LPS. The L2FC (Log 2-fold change) states the average difference in gene expression between both treatments. Positive L2FC values denote upregulation by LPS treatment, negative values downregulation. Wald test from DESeq2 was used to calculate the significance of the change in the expression. The adjusted p-values take the number of tested genes into account, the threshold for the adjusted p-value was 0.05.

gene name	Ensembl gene ID	L2FC	p-value	adusted p-value
LAMC2	ENSG00000058085	2,175	1,73E-211	2,50E-207
EBI3	ENSG00000105246	2,426	1,30E-60	9,40E-57
CXCL6	ENSG00000124875	1,813	1,84E-58	8,86E-55
SOD2	ENSG00000112096	1,027	2,11E-57	7,64E-54
CCL2	ENSG00000108691	1,201	6,20E-57	1,79E-53
UBD	ENSG00000226898	2,737	4,26E-56	1,03E-52
CFB	ENSG00000242335	1,832	3,80E-54	7,85E-51
MMP10	ENSG00000166670	1,176	2,23E-52	4,04E-49
CTSS	ENSG00000163131	1,296	2,29E-41	3,69E-38
IL32	ENSG00000008517	1,326	5,27E-36	7,63E-33
CTSK	ENSG00000143387	1,404	1,42E-35	1,87E-32
POU2F2	ENSG00000028277	1,233	3,86E-35	4,65E-32
LTB	ENSG00000223448	1,341	2,37E-34	2,64E-31
S100A3	ENSG00000188015	2,189	1,39E-33	1,44E-30
IGFBP3	ENSG00000146674	-0,846	3,62E-31	3,49E-28
PAPLN	ENSG00000100767	1,617	2,35E-29	2,13E-26
HLA-B	ENSG00000206450	1,221	5,52E-25	4,70E-22
ISG20	ENSG00000172183	0,842	2,84E-24	2,29E-21
CXCL1	ENSG00000163739	1,075	6,17E-23	4,70E-20
CNTNAP1	ENSG00000108797	0,572	1,34E-22	9,73E-20
CXCL3	ENSG00000163734	0,929	2,43E-22	1,67E-19
PLA2G4C	ENSG00000105499	0,885	6,16E-22	4,05E-19
IFI27	ENSG00000275214	0,615	1,36E-21	8,58E-19
MX1	ENSG00000157601	1,110	1,53E-20	9,21E-18
ANO9	ENSG00000185101	4,656	2,14E-20	1,24E-17
CXCL5	ENSG00000163735	1,905	5,18E-20	2,88E-17
ICAM1	ENSG00000090339	0,723	1,24E-18	6,64E-16
PSMB9	ENSG00000243958	0,719	2,21E-16	1,14E-13
IL4I1	ENSG00000104951	0,729	1,24E-15	6,21E-13
CXCL2	ENSG00000081041	1,059	2,09E-14	1,01E-11
SLC7A2	ENSG00000003989	0,634	4,06E-14	1,89E-11
THSD4	ENSG00000187720	0,353	4,20E-14	1,90E-11
MAMDC2	ENSG00000278608	0,919	8,41E-14	3,69E-11
IFI6	ENSG00000126709	0,620	1,71E-13	7,28E-11
NEURL1B	ENSG00000214357	-0,767	1,85E-13	7,63E-11
TFPI2	ENSG00000105825	0,766	2,66E-13	1,07E-10
AC139530.2	NA	-30,000	9,87E-13	3,86E-10
TNFRSF9	ENSG00000049249	1,451	1,08E-12	4,10E-10
LAMP3	ENSG00000078081	0,608	4,93E-12	1,83E-09
UBE2L6	ENSG00000156587	0,403	1,16E-11	4,19E-09
OAS2	ENSG00000111335	1,100	2,43E-11	8,58E-09
PSMB8	ENSG00000230669	0,433	3,00E-11	1,03E-08
CCL20	ENSG00000115009	1,110	4,08E-11	1,37E-08
CYB5R2	ENSG00000166394	0,696	5,67E-11	1,86E-08
GPSM2	ENSG00000121957	-0,652	7,28E-11	2,34E-08

CTHRC1	ENSG00000164932	0,593	1,00E-10	3,15E-08
P2RX4	ENSG00000135124	0,371	1,44E-10	4,40E-08
TNC	ENSG00000041982	3,090	1,46E-10	4,40E-08
CXCL8	ENSG00000169429	0,851	1,77E-10	5,21E-08
STAP2	ENSG00000178078	0,498	2,27E-10	6,56E-08
CSF2	ENSG00000164400	1,656	3,48E-10	9,87E-08
ARHGDIG	ENSG00000242173	0,656	4,24E-10	1,17E-07
C2CD4A	ENSG00000198535	0,895	4,27E-10	1,17E-07
PRICKLE1	ENSG00000139174	-0,558	6,19E-10	1,66E-07
CAV1	ENSG00000105974	-0,380	7,33E-10	1,93E-07
CDKN3	ENSG00000100526	-0,614	8,83E-10	2,21E-07
KIF20A	ENSG00000112984	-0,583	8,86E-10	2,21E-07
METTL7A	ENSG00000185432	-0,478	8,64E-10	2,21E-07
CYP51A1	ENSG00000001630	-0,352	1,73E-09	4,24E-07
STARD10	ENSG00000214530	0,569	1,99E-09	4,79E-07
VWA1	ENSG00000179403	0,476	3,39E-09	8,05E-07
MX2	ENSG00000183486	2,548	3,69E-09	8,60E-07
APOL1	ENSG00000100342	0,388	5,39E-09	1,24E-06
TAPBP	ENSG00000206281	0,477	5,53E-09	1,25E-06
GP1BB	ENSG00000203618	-22,691	6,22E-09	1,38E-06
DHRS3	ENSG00000162496	0,429	6,82E-09	1,50E-06
CEBPD	ENSG00000221869	0,785	7,57E-09	1,63E-06
PLA1A	ENSG00000144837	1,472	7,81E-09	1,66E-06
WASF3	ENSG00000132970	-0,313	9,19E-09	1,93E-06
LYPD6	ENSG00000187123	0,469	1,20E-08	2,47E-06
CAPN2	ENSG00000162909	-0,289	1,47E-08	2,95E-06
HLA-A	ENSG00000227715	0,395	1,47E-08	2,95E-06
HMGCS1	ENSG00000112972	-0,427	1,83E-08	3,63E-06
BUB1	ENSG00000169679	-0,492	1,96E-08	3,83E-06
SELENOM	ENSG00000198832	0,545	1,98E-08	3,83E-06
F2RL1	ENSG00000164251	0,380	2,29E-08	4,36E-06
BST1	ENSG00000109743	0,394	2,56E-08	4,81E-06
PCK2	ENSG00000285241	0,426	2,61E-08	4,84E-06
TNFSF18	ENSG00000120337	-0,583	3,14E-08	5,75E-06
DENND2B	ENSG00000166444	0,984	3,20E-08	5,79E-06
AL451062.4	NA	-23,299	3,34E-08	5,98E-06
UBA7	ENSG00000182179	0,446	3,41E-08	6,02E-06
ALOX5AP	ENSG00000132965	1,587	3,68E-08	6,41E-06
DLGAP5	ENSG00000126787	-0,621	4,30E-08	7,41E-06
CRYBG1	ENSG00000112297	-0,333	4,95E-08	8,42E-06
PRSS12	ENSG00000164099	1,198	5,39E-08	9,07E-06
LIPG	ENSG00000101670	0,513	5,53E-08	9,19E-06
SLC15A3	ENSG00000110446	0,517	5,73E-08	9,43E-06
ICOSLG	ENSG00000160223	0,533	7,11E-08	1,16E-05
OAS1	ENSG00000089127	0,519	8,71E-08	1,40E-05
CCNB2	ENSG00000157456	-0,480	9,09E-08	1,45E-05
AL358472.7	NA	20,830	9,30E-08	1,46E-05
ACBD7	ENSG00000176244	-0,775	1,04E-07	1,62E-05
MEST	ENSG00000106484	-0,435	1,21E-07	1,86E-05
JAK3	ENSG00000105639	0,743	1,22E-07	1,86E-05
ALDH1A1	ENSG00000165092	-0,403	1,34E-07	2,02E-05
CRTAC1	ENSG00000095713	0,499	1,67E-07	2,49E-05
OCIAD2	ENSG00000145247	0,437	1,74E-07	2,56E-05

CD69	ENSG00000110848	0,949	1,88E-07	2,75E-05
MKI67	ENSG00000148773	-0,525	1,98E-07	2,87E-05
FSTL3	ENSG00000070404	0,444	2,37E-07	3,39E-05
ADAMTS18	ENSG00000140873	-0,551	2,60E-07	3,59E-05
CIT	ENSG00000122966	-0,468	2,53E-07	3,59E-05
TMEM120A	ENSG00000189077	0,427	2,59E-07	3,59E-05
TNFRSF4	ENSG00000186827	0,959	2,61E-07	3,59E-05
DHH	ENSG00000139549	-0,464	3,68E-07	5,02E-05
PRR11	ENSG00000068489	-0,326	3,87E-07	5,24E-05
SERPINA3	ENSG00000196136	2,440	4,12E-07	5,52E-05
KIF12	ENSG00000136883	0,697	6,16E-07	8,18E-05
PLCG2	ENSG00000197943	1,263	6,57E-07	8,65E-05
PSME2	ENSG00000284889	0,443	6,76E-07	8,81E-05
BDKRB2	ENSG00000168398	0,931	8,27E-07	1,07E-04
ACSS1	ENSG00000154930	0,279	8,67E-07	1,11E-04
MAP2K6	ENSG00000108984	-0,373	8,88E-07	1,13E-04
CCL15-CCL14	ENSG00000282521	-0,416	9,40E-07	1,18E-04
MMP19	ENSG00000123342	0,573	1,04E-06	1,29E-04
ACE	ENSG00000159640	-0,620	1,11E-06	1,37E-04
ZCCHC2	ENSG00000141664	-0,507	1,16E-06	1,42E-04
PAQR7	ENSG00000182749	0,277	1,18E-06	1,42E-04
RASA4B	ENSG00000170667	0,567	1,18E-06	1,42E-04
CCNB1	ENSG00000134057	-0,496	1,20E-06	1,43E-04
PLAAT4	ENSG00000133321	0,728	1,30E-06	1,54E-04
TAPBPL	ENSG00000139192	0,812	1,37E-06	1,61E-04
LYVE1	ENSG00000133800	-0,703	1,52E-06	1,78E-04
APOD	ENSG00000189058	0,469	1,76E-06	2,04E-04
ZC3H12A	ENSG00000163874	0,514	1,86E-06	2,14E-04
CEP55	ENSG00000138180	-0,538	1,94E-06	2,21E-04
ITGAV	ENSG00000138448	0,523	2,09E-06	2,37E-04
ARHGEF19	ENSG00000142632	0,356	2,35E-06	2,64E-04
MAP3K6	ENSG00000142733	0,371	2,50E-06	2,76E-04
SEMA3G	ENSG00000010319	0,417	2,49E-06	2,76E-04
POSTN	ENSG00000133110	-0,702	2,60E-06	2,85E-04
GXYLT2	ENSG00000172986	1,226	2,62E-06	2,85E-04
AC087632.2	NA	-1,660	3,01E-06	3,23E-04
ZDHHC13	ENSG00000177054	-0,297	3,01E-06	3,23E-04
NUCKS1	ENSG00000069275	-0,368	3,07E-06	3,27E-04
CKAP2	ENSG00000136108	-0,456	3,21E-06	3,39E-04
ALDH1A2	ENSG00000128918	-0,356	3,38E-06	3,55E-04
ZNF791	ENSG00000173875	-0,512	3,74E-06	3,90E-04
AKAP12	ENSG00000131016	0,272	4,09E-06	4,23E-04
MTMR10	ENSG00000277086	-0,375	4,67E-06	4,79E-04
AL109918.1	NA	-0,486	4,70E-06	4,80E-04
CERS1	ENSG00000223802	0,381	4,84E-06	4,90E-04
C11orf96	ENSG00000187479	0,453	5,27E-06	5,30E-04
PLCG1	ENSG00000124181	0,243	5,57E-06	5,56E-04
MYRIP	ENSG00000170011	-0,347	5,87E-06	5,81E-04
CXADR	ENSG00000154639	-0,411	6,06E-06	5,93E-04
RASA4	ENSG00000105808	0,440	6,03E-06	5,93E-04
INCENP	ENSG00000149503	-0,443	6,36E-06	6,18E-04
ELMOD1	ENSG00000110675	-0,476	6,88E-06	6,64E-04
KNL1	ENSG00000137812	-0,713	7,35E-06	6,93E-04

MSMP	ENSG00000215183	-0,370	7,33E-06	6,93E-04
SAMD14	ENSG00000167100	0,369	7,29E-06	6,93E-04
SDC4	ENSG00000124145	0,352	7,37E-06	6,93E-04
MCFD2	ENSG00000180398	-0,277	7,55E-06	7,05E-04
BST2	ENSG00000130303	0,315	7,71E-06	7,15E-04
MYZAP	ENSG00000263155	-0,413	8,20E-06	7,55E-04
CENPF	ENSG00000117724	-0,557	8,47E-06	7,76E-04
HMMR	ENSG00000072571	-0,747	8,70E-06	7,92E-04
TSPAN13	ENSG00000106537	0,390	9,00E-06	8,14E-04
ASAP1	ENSG00000153317	-0,339	9,46E-06	8,50E-04
PIGK	ENSG00000142892	-0,369	9,79E-06	8,75E-04
PRCP	ENSG00000137509	0,212	1,02E-05	9,10E-04
PDCD6IP	ENSG00000170248	-0,363	1,11E-05	9,81E-04
LRFN4	ENSG00000173621	0,375	1,13E-05	9,94E-04
ACAT2	ENSG00000120437	-0,299	1,15E-05	9,95E-04
FXYD6	ENSG00000137726	0,656	1,15E-05	9,95E-04
R3HDM1	ENSG00000048991	-0,468	1,16E-05	1,00E-03
TNFRSF14	ENSG00000273936	0,388	1,20E-05	1,03E-03
AC087721.2	NA	-0,375	1,31E-05	1,11E-03
KIT	ENSG00000157404	0,262	1,33E-05	1,13E-03
IL27RA	ENSG00000288185	0,362	1,36E-05	1,14E-03
NEK2	ENSG00000117650	-0,489	1,35E-05	1,14E-03
SYNJ2	ENSG00000078269	0,160	1,36E-05	1,14E-03
TMPO	ENSG00000120802	-0,320	1,40E-05	1,16E-03
DEPDC1B	ENSG00000035499	-0,341	1,45E-05	1,19E-03
SEMA7A	ENSG00000288455	0,515	1,46E-05	1,19E-03
IRF9	ENSG00000285048	0,498	1,55E-05	1,26E-03
MTMR11	ENSG00000014914	0,364	1,57E-05	1,27E-03
MGARP	ENSG00000137463	-0,476	1,62E-05	1,31E-03
KCNJ15	ENSG00000157551	-0,746	1,74E-05	1,39E-03
MGME1	ENSG00000125871	-0,250	1,79E-05	1,42E-03
BORA	ENSG00000136122	-0,605	1,87E-05	1,48E-03
CENPA	ENSG00000115163	-0,407	1,95E-05	1,53E-03
ERMP1	ENSG00000099219	-0,267	1,95E-05	1,53E-03
ITGB4	ENSG00000132470	-0,576	2,06E-05	1,61E-03
CBR3	ENSG00000159231	0,357	2,09E-05	1,62E-03
PPL	ENSG00000118898	-0,801	2,30E-05	1,77E-03
SPTLC1P1	ENSG00000230397	-0,705	2,49E-05	1,91E-03
HSPE1-MOB4	ENSG00000270757	-0,483	2,51E-05	1,91E-03
ASPM	ENSG00000066279	-0,784	2,55E-05	1,93E-03
COBLL1	ENSG00000082438	-0,381	2,60E-05	1,95E-03
ULBP2	ENSG00000131015	0,338	2,59E-05	1,95E-03
CX3CL1	ENSG00000006210	0,553	2,63E-05	1,96E-03
RAB14	ENSG00000119396	-0,247	2,72E-05	2,01E-03
TOP2A	ENSG00000131747	-0,435	2,73E-05	2,01E-03
AC091951.4	NA	2,510	3,21E-05	2,35E-03
DPP4	ENSG00000197635	-0,347	3,23E-05	2,35E-03
LAMB3	ENSG00000196878	0,446	3,26E-05	2,35E-03
NFKBIZ	ENSG00000144802	0,541	3,24E-05	2,35E-03
NUF2	ENSG00000143228	-0,646	3,24E-05	2,35E-03
ARL5A	ENSG00000162980	-0,701	3,35E-05	2,40E-03
ABCA8	ENSG00000141338	-0,677	3,54E-05	2,53E-03
DIAPH3	ENSG00000139734	-0,353	3,57E-05	2,53E-03

FAHD2CP	ENSG00000231584	0,381	3,69E-05	2,59E-03
FP565260.3	NA	0,391	3,68E-05	2,59E-03
PSMB10	ENSG00000205220	0,328	3,84E-05	2,68E-03
TMEM121	ENSG00000184986	0,381	3,87E-05	2,69E-03
IFITM1	ENSG00000185885	0,366	4,00E-05	2,77E-03
SYNJ1	ENSG00000159082	-0,312	4,26E-05	2,94E-03
EMCN	ENSG00000164035	-0,524	4,30E-05	2,95E-03
IFIT1	ENSG00000185745	0,820	4,33E-05	2,95E-03
CCL14	ENSG00000277236	-0,431	4,57E-05	3,11E-03
VAPA	ENSG00000101558	-0,205	4,75E-05	3,21E-03
ACKR4	ENSG00000129048	-0,562	4,85E-05	3,26E-03
HMGCR	ENSG00000113161	-0,321	4,93E-05	3,30E-03
APAF1	ENSG00000120868	-0,294	5,05E-05	3,35E-03
CLDN14	ENSG00000159261	0,877	5,05E-05	3,35E-03
FBXO32	ENSG00000156804	0,355	5,18E-05	3,42E-03
CSF3	ENSG00000108342	1,066	5,63E-05	3,71E-03
CENPE	ENSG00000138778	-0,750	5,78E-05	3,78E-03
CPLANE2	ENSG00000132881	0,390	5,88E-05	3,83E-03
CAMTA2	ENSG00000108509	0,290	6,00E-05	3,88E-03
SLC22A31	ENSG00000259803	0,695	6,00E-05	3,88E-03
IFI35	ENSG00000068079	0,380	6,14E-05	3,95E-03
ZNF365	ENSG00000138311	0,517	6,32E-05	4,05E-03
PSME1	ENSG00000284916	0,266	6,45E-05	4,11E-03
GDF15	ENSG00000130513	0,428	6,56E-05	4,16E-03
HIBCH	ENSG00000198130	-0,436	6,70E-05	4,24E-03
ACHE	ENSG00000087085	0,801	6,78E-05	4,26E-03
FTH1	ENSG00000167996	0,268	6,87E-05	4,30E-03
RPS6KL1	ENSG00000198208	0,352	6,97E-05	4,35E-03
PTK7	ENSG00000112655	0,269	7,23E-05	4,49E-03
TNFRSF6B	ENSG00000243509	0,338	7,31E-05	4,52E-03
C17orf107	ENSG00000205710	0,459	7,81E-05	4,81E-03
NECTIN3	ENSG00000177707	-0,470	8,03E-05	4,92E-03
RNASE1	ENSG00000129538	-0,276	8,27E-05	5,05E-03
AFF1	ENSG00000172493	-0,327	8,44E-05	5,05E-03
GLRX	ENSG00000173221	-0,240	8,45E-05	5,05E-03
GRASP	NA	0,197	8,45E-05	5,05E-03
LPCAT2	ENSG00000087253	-0,362	8,40E-05	5,05E-03
NFKBIA	ENSG00000100906	0,272	8,34E-05	5,05E-03
INSIG1	ENSG00000186480	-0,278	9,05E-05	5,37E-03
TMOD1	ENSG00000136842	0,496	9,02E-05	5,37E-03
B3GALT4	ENSG00000235155	0,398	9,28E-05	5,43E-03
CCNYL1	ENSG00000163249	-0,395	9,26E-05	5,43E-03
MFAP2	ENSG00000117122	0,276	9,30E-05	5,43E-03
SYNGR3	ENSG00000127561	0,367	9,20E-05	5,43E-03
HTR2B	ENSG00000135914	-0,426	9,46E-05	5,50E-03
ADAM12	ENSG00000148848	1,044	9,52E-05	5,51E-03
MEF2C	ENSG00000081189	-0,347	9,70E-05	5,59E-03
SAPCD2	ENSG00000186193	-0,377	9,74E-05	5,60E-03
MS4A6A	ENSG00000110077	0,466	9,89E-05	5,66E-03
PLTP	ENSG00000100979	0,355	9,95E-05	5,67E-03
CCNA2	ENSG00000145386	-0,400	1,01E-04	5,75E-03
DPP3	ENSG00000254986	0,281	1,02E-04	5,75E-03
P2RY6	ENSG00000171631	1,229	1,03E-04	5,79E-03

MAP6	ENSG00000171533	0,992	1,05E-04	5,88E-03
TCEAL7	ENSG00000182916	0,328	1,05E-04	5,88E-03
TGFBR3L	ENSG00000260001	0,467	1,12E-04	6,18E-03
TMEM132A	ENSG00000006118	0,351	1,11E-04	6,18E-03
WDFY3	ENSG00000163625	-0,381	1,12E-04	6,18E-03
EPSTI1	ENSG00000133106	0,802	1,13E-04	6,22E-03
RND1	ENSG00000172602	0,401	1,15E-04	6,28E-03
HLA-H	ENSG00000231904	0,356	1,15E-04	6,30E-03
MSMO1	ENSG00000052802	-0,536	1,16E-04	6,33E-03
RNMT	ENSG00000101654	-0,425	1,17E-04	6,33E-03
MPHOSPH9	ENSG00000051825	-0,464	1,19E-04	6,44E-03
RAB7B	ENSG00000276600	1,191	1,20E-04	6,46E-03
AMPH	ENSG00000078053	0,488	1,23E-04	6,61E-03
HAS2	ENSG00000170961	1,050	1,30E-04	6,88E-03
KIF4A	ENSG00000090889	-0,318	1,29E-04	6,88E-03
SNX22	ENSG00000157734	0,983	1,30E-04	6,88E-03
AQP1	ENSG00000240583	-1,028	1,33E-04	6,98E-03
H2AC6	ENSG00000180573	-0,697	1,33E-04	6,98E-03
CASP1	ENSG00000137752	0,488	1,34E-04	6,99E-03
COTL1	ENSG00000103187	0,280	1,35E-04	6,99E-03
GSDMD	ENSG00000278718	0,274	1,35E-04	6,99E-03
KIF14	ENSG00000118193	-0,564	1,35E-04	6,99E-03
QTRT2	ENSG00000151576	-0,279	1,34E-04	6,99E-03
EFCAB14	ENSG00000159658	-0,243	1,40E-04	7,20E-03
IER3	ENSG00000235030	0,270	1,40E-04	7,20E-03
RASD1	ENSG00000108551	0,316	1,49E-04	7,60E-03
AOX1	ENSG00000138356	0,614	1,55E-04	7,85E-03
FRY	ENSG00000073910	-0,171	1,55E-04	7,85E-03
NBPF10	ENSG00000271425	-0,326	1,55E-04	7,85E-03
STEAP1	ENSG00000164647	-0,346	1,56E-04	7,86E-03
SULT1B1	ENSG00000173597	-0,498	1,58E-04	7,96E-03
OIP5	ENSG00000104147	-0,352	1,61E-04	8,05E-03
ANKH	ENSG00000154122	0,290	1,63E-04	8,12E-03
TMEM54	ENSG00000121900	0,266	1,64E-04	8,15E-03
SLC1A4	ENSG00000115902	0,234	1,68E-04	8,33E-03
IL11	ENSG00000095752	0,997	1,69E-04	8,34E-03
CGNL1	ENSG00000128849	-0,198	1,73E-04	8,48E-03
NRG1	ENSG00000157168	-0,397	1,73E-04	8,48E-03
RBBP9	ENSG00000089050	-0,332	1,74E-04	8,48E-03
CKS2	ENSG00000123975	-0,336	1,83E-04	8,89E-03
HLA-C	ENSG00000206435	0,313	1,83E-04	8,89E-03
TNIP3	ENSG00000050730	1,038	1,84E-04	8,90E-03
ADAM32	ENSG00000275594	-1,239	1,85E-04	8,91E-03
MYO5A	ENSG00000197535	-0,276	1,85E-04	8,91E-03
ACER3	ENSG00000078124	-0,213	1,97E-04	9,46E-03
RELB	ENSG00000104856	0,353	2,00E-04	9,55E-03
GBP4	ENSG00000162654	0,310	2,02E-04	9,61E-03
RAB11FIP2	ENSG00000107560	-0,544	2,02E-04	9,61E-03
SURF1	ENSG00000280627	0,234	2,04E-04	9,64E-03
MYH10	ENSG00000133026	-0,217	2,09E-04	9,83E-03
PIMREG	ENSG00000129195	-0,446	2,09E-04	9,83E-03
IL1A	ENSG00000115008	0,507	2,18E-04	1,02E-02
GMPPA	ENSG00000144591	0,312	2,20E-04	1,03E-02

ARL6IP1	ENSG00000170540	-0,280	2,22E-04	1,03E-02
CNN3	ENSG00000117519	-0,234	2,24E-04	1,04E-02
GALNT18	ENSG00000110328	0,494	2,25E-04	1,04E-02
FNBP1	ENSG00000187239	-0,269	2,28E-04	1,05E-02
PARP10	ENSG00000178685	0,319	2,28E-04	1,05E-02
MLIP	ENSG00000146147	-0,301	2,45E-04	1,12E-02
TM4SF18	ENSG00000163762	-0,366	2,45E-04	1,12E-02
UBR4	ENSG00000127481	0,376	2,47E-04	1,12E-02
RMDN2	ENSG00000115841	-0,550	2,48E-04	1,13E-02
HOXB9	ENSG00000170689	0,395	2,52E-04	1,14E-02
ARSA	ENSG00000100299	0,292	2,55E-04	1,15E-02
SOCS3	ENSG00000184557	0,302	2,55E-04	1,15E-02
FBLN5	ENSG00000140092	0,404	2,58E-04	1,15E-02
TSPAN5	ENSG00000168785	-0,195	2,58E-04	1,15E-02
RSAD1	ENSG00000136444	0,197	2,64E-04	1,18E-02
ARHGAP11A	ENSG00000275568	-0,412	2,65E-04	1,18E-02
ATP5F1D	ENSG00000099624	0,341	2,66E-04	1,18E-02
NSD2	ENSG00000109685	-0,239	2,68E-04	1,18E-02
ISG15	ENSG00000187608	0,345	2,81E-04	1,23E-02
PBK	ENSG00000168078	-0,449	2,82E-04	1,23E-02
LATS2	ENSG00000150457	-0,211	2,83E-04	1,24E-02
AGRN	ENSG00000188157	0,368	2,93E-04	1,28E-02
TSSC4	ENSG00000184281	0,419	2,95E-04	1,28E-02
PMM1	ENSG00000100417	0,341	3,00E-04	1,30E-02
H2AC11	ENSG00000196787	-0,650	3,06E-04	1,32E-02
EML2	ENSG00000125746	0,311	3,14E-04	1,35E-02
PCDH1	ENSG00000156453	0,269	3,14E-04	1,35E-02
NCKAP5	ENSG00000176771	0,847	3,16E-04	1,35E-02
TSPAN11	ENSG00000110900	-0,360	3,21E-04	1,37E-02
CEP70	ENSG00000114107	-0,520	3,27E-04	1,39E-02
CKAP5	ENSG00000175216	-0,220	3,32E-04	1,40E-02
HSPB8	ENSG00000152137	0,347	3,31E-04	1,40E-02
AHNAK	ENSG00000124942	-0,388	3,34E-04	1,41E-02
CCM2L	ENSG00000101331	-0,325	3,38E-04	1,42E-02
DUSP4	ENSG00000120875	-0,269	3,41E-04	1,43E-02
SULT1E1	ENSG00000109193	-0,482	3,41E-04	1,43E-02
TCIRG1	ENSG00000110719	0,271	3,43E-04	1,43E-02
FXYD2	ENSG00000137731	0,734	3,45E-04	1,43E-02
UBN2	ENSG00000157741	-0,561	3,47E-04	1,44E-02
SPAG5	ENSG00000076382	-0,278	3,49E-04	1,44E-02
THBS2	ENSG00000186340	0,836	3,48E-04	1,44E-02
ZNF800	ENSG00000048405	-0,353	3,52E-04	1,45E-02
SGO2	ENSG00000163535	-0,656	3,64E-04	1,49E-02
GFPT2	ENSG00000131459	0,367	3,69E-04	1,51E-02
APLN	ENSG00000171388	-0,407	3,81E-04	1,55E-02
ARHGAP11B	ENSG00000274734	-0,429	3,86E-04	1,57E-02
JUNB	ENSG00000171223	0,259	3,87E-04	1,57E-02
BPGM	ENSG00000172331	0,259	3,89E-04	1,57E-02
CDH15	ENSG00000129910	0,509	3,90E-04	1,57E-02
MTMR7	ENSG00000003987	0,867	3,93E-04	1,58E-02
ST6GALNAC2	ENSG00000070731	0,756	3,98E-04	1,60E-02
AC241640.1	NA	-1,539	4,06E-04	1,62E-02
SFRP1	ENSG00000104332	-0,311	4,07E-04	1,62E-02

LIG1	ENSG00000105486	0,274	4,10E-04	1,63E-02
SLC7A7	ENSG00000155465	0,298	4,23E-04	1,68E-02
CXCR4	ENSG00000121966	0,237	4,25E-04	1,68E-02
GBGT1	ENSG00000148288	0,343	4,36E-04	1,72E-02
SESN3	ENSG00000149212	-0,641	4,37E-04	1,72E-02
MAPK1	ENSG00000100030	-0,294	4,38E-04	1,72E-02
AC079594.2	NA	-0,631	4,42E-04	1,73E-02
FMNL1	ENSG00000184922	0,395	4,45E-04	1,74E-02
HIVEP1	ENSG00000095951	-0,366	4,48E-04	1,74E-02
PLEKHN1	ENSG00000187583	0,492	4,49E-04	1,74E-02
RBL2	ENSG00000103479	-0,287	4,48E-04	1,74E-02
PTGS2	ENSG00000073756	0,553	4,55E-04	1,76E-02
WNK3	ENSG00000196632	-0,654	4,59E-04	1,77E-02
NMNAT2	ENSG00000157064	0,987	4,62E-04	1,77E-02
TMSB15A	ENSG00000158164	-0,521	4,64E-04	1,77E-02
WASF1	ENSG00000112290	-0,307	4,63E-04	1,77E-02
PTGES3L	ENSG00000267060	1,020	4,69E-04	1,79E-02
GMFG	ENSG00000130755	0,214	4,74E-04	1,80E-02
HP1BP3	ENSG00000127483	-0,314	4,79E-04	1,81E-02
SLC35C2	ENSG00000080189	0,269	4,88E-04	1,84E-02
SLC27A3	ENSG00000263163	0,380	4,93E-04	1,86E-02
CFAP69	ENSG00000105792	-0,585	4,96E-04	1,86E-02
ZBTB42	ENSG00000179627	0,284	4,98E-04	1,87E-02
CPAMD8	ENSG00000160111	0,499	5,08E-04	1,88E-02
MAP1LC3A	ENSG00000101460	0,328	5,06E-04	1,88E-02
MT1L	ENSG00000260549	0,355	5,05E-04	1,88E-02
SMAD5	ENSG00000113658	-0,472	5,07E-04	1,88E-02
SMG1P3	ENSG00000180747	-0,283	5,13E-04	1,90E-02
DAW1	ENSG00000123977	-0,338	5,15E-04	1,90E-02
SLC38A5	ENSG00000017483	0,391	5,18E-04	1,91E-02
ACBD4	ENSG00000181513	0,278	5,27E-04	1,93E-02
SUV39H2	ENSG00000152455	-0,517	5,28E-04	1,93E-02
CPA4	ENSG00000128510	-0,847	5,34E-04	1,94E-02
MROH6	ENSG00000277781	0,678	5,31E-04	1,94E-02
NFATC4	ENSG00000285485	0,371	5,35E-04	1,94E-02
TM7SF3	ENSG00000064115	-0,245	5,34E-04	1,94E-02
UPP1	ENSG00000183696	0,232	5,41E-04	1,96E-02
ADGRB2	ENSG00000121753	0,324	5,56E-04	2,01E-02
ATOH8	ENSG00000168874	0,364	5,61E-04	2,02E-02
KNSTRN	ENSG00000128944	-0,257	5,71E-04	2,04E-02
ODF3B	ENSG00000177989	0,753	5,69E-04	2,04E-02
SERPINB9	ENSG00000170542	0,176	5,69E-04	2,04E-02
PTP4A1	ENSG00000112245	-0,335	5,88E-04	2,09E-02
TMC6	ENSG00000141524	0,276	5,88E-04	2,09E-02
SPTA1	ENSG00000163554	-1,154	5,92E-04	2,10E-02
SENP1	ENSG00000079387	-0,270	5,95E-04	2,11E-02
PIK3CD	ENSG00000171608	0,296	6,11E-04	2,16E-02
SPHK1	ENSG00000176170	0,285	6,15E-04	2,16E-02
TSPAN8	ENSG00000127324	-0,637	6,15E-04	2,16E-02
CD34	ENSG00000174059	-0,226	6,22E-04	2,18E-02
PLAC8	ENSG00000145287	-0,413	6,26E-04	2,19E-02
E2F1	ENSG00000101412	0,419	6,32E-04	2,20E-02
AMOT	ENSG00000126016	-0,550	6,38E-04	2,22E-02

C2CD2	ENSG00000157617	-0,279	6,60E-04	2,28E-02
CST1	ENSG00000170373	0,592	6,60E-04	2,28E-02
SAT1	ENSG00000130066	0,334	6,60E-04	2,28E-02
EFNA2	ENSG00000099617	0,374	6,66E-04	2,30E-02
ANGEL2	ENSG00000174606	-0,371	6,77E-04	2,33E-02
MCM9	ENSG00000111877	-0,297	6,81E-04	2,33E-02
POLD1	ENSG00000062822	0,320	6,82E-04	2,33E-02
SH3TC2	ENSG00000169247	-0,555	6,88E-04	2,35E-02
PHACTR4	ENSG00000204138	-0,233	6,93E-04	2,36E-02
HNRNPLL	ENSG00000143889	-0,211	6,99E-04	2,37E-02
MYO5C	ENSG00000128833	-0,322	7,01E-04	2,37E-02
TREX1	ENSG00000213689	0,275	7,02E-04	2,37E-02
LPAR1	ENSG00000198121	-0,631	7,06E-04	2,38E-02
DNM1P47	ENSG00000259660	-0,477	7,12E-04	2,40E-02
ARHGAP17	ENSG00000288353	-0,465	7,24E-04	2,43E-02
FBXO15	ENSG00000141665	-1,172	7,23E-04	2,43E-02
MMRN1	ENSG00000138722	-0,492	7,26E-04	2,43E-02
TNFSF15	ENSG00000181634	0,218	7,37E-04	2,46E-02
OCLN	ENSG00000273814	-0,336	7,39E-04	2,46E-02
CDK19	ENSG00000155111	-0,377	7,45E-04	2,47E-02
GALT	ENSG00000213930	0,230	7,58E-04	2,51E-02
C2CD4B	ENSG00000205502	0,558	7,68E-04	2,54E-02
IFI44L	ENSG00000137959	0,720	7,78E-04	2,56E-02
CCDC28B	ENSG00000160050	0,254	7,83E-04	2,57E-02
ADM2	ENSG00000128165	0,487	7,92E-04	2,59E-02
GAS2L3	ENSG00000139354	-0,436	7,90E-04	2,59E-02
BATF3	ENSG00000123685	0,662	7,95E-04	2,60E-02
ARNTL	ENSG00000133794	-0,285	8,03E-04	2,61E-02
TPPP3	ENSG00000159713	0,496	8,03E-04	2,61E-02
IPO5	ENSG00000065150	-0,156	8,09E-04	2,63E-02
GPNMB	ENSG00000136235	-0,996	8,15E-04	2,64E-02
IL3RA	ENSG00000185291	0,238	8,30E-04	2,67E-02
PDE3A	ENSG00000172572	-0,239	8,29E-04	2,67E-02
SCUBE3	ENSG00000146197	-0,411	8,38E-04	2,69E-02
ADAMTS12	ENSG00000281690	-0,546	8,53E-04	2,74E-02
PIK3CG	ENSG00000105851	-0,918	8,54E-04	2,74E-02
THOC3	ENSG00000051596	-0,528	8,70E-04	2,78E-02
COL4A2	ENSG00000134871	0,432	8,84E-04	2,82E-02
BLOC1S3	ENSG00000189114	0,447	9,02E-04	2,87E-02
APOBEC3G	ENSG00000239713	0,249	9,11E-04	2,89E-02
DHX58	ENSG00000108771	0,330	9,19E-04	2,91E-02
SGO1	ENSG00000129810	-0,365	9,31E-04	2,94E-02
PIK3R3	ENSG00000117461	-0,301	9,33E-04	2,94E-02
MOBP	ENSG00000168314	0,777	9,37E-04	2,95E-02
RACGAP1	ENSG00000161800	-0,327	9,45E-04	2,97E-02
C19orf47	ENSG00000160392	0,357	9,56E-04	2,99E-02
DHCR7	ENSG00000172893	-0,348	9,60E-04	3,00E-02
FHL3	ENSG00000183386	0,276	9,60E-04	3,00E-02
MELTF	ENSG00000163975	0,373	9,63E-04	3,00E-02
CCNE1	ENSG00000105173	0,263	9,67E-04	3,00E-02
CFAP410	ENSG00000160226	0,304	9,90E-04	3,05E-02
LRRC3	ENSG00000160233	0,273	9,89E-04	3,05E-02
PTTG1	ENSG00000164611	-0,362	9,88E-04	3,05E-02

SLC26A4	ENSG00000091137	-0,719	9,91E-04	3,05E-02
NDE1	ENSG00000275911	-0,219	1,01E-03	3,10E-02
TNFRSF21	ENSG00000146072	0,172	1,01E-03	3,10E-02
MMP28	ENSG00000278843	-1,256	1,02E-03	3,11E-02
WASHC5	ENSG00000164961	-0,200	1,02E-03	3,11E-02
NUDT22	ENSG00000149761	0,284	1,03E-03	3,13E-02
SIPA1	ENSG00000213445	0,279	1,03E-03	3,13E-02
PPP3CA	ENSG00000138814	-0,232	1,04E-03	3,15E-02
MFSD3	ENSG00000167700	0,235	1,04E-03	3,15E-02
TYMP	ENSG00000025708	0,669	1,04E-03	3,16E-02
DNAH5	ENSG00000039139	0,375	1,05E-03	3,16E-02
AC005363.1	NA	6,479	1,06E-03	3,18E-02
IGF2BP3	ENSG00000136231	-0,313	1,06E-03	3,19E-02
FEM1B	ENSG00000169018	-0,299	1,07E-03	3,20E-02
RALB	ENSG00000144118	-0,165	1,07E-03	3,20E-02
ASPHD2	ENSG00000128203	0,209	1,08E-03	3,21E-02
GALNT12	ENSG00000119514	0,317	1,08E-03	3,21E-02
RPL23AP87	ENSG00000232938	2,313	1,08E-03	3,21E-02
COMMD3-BMI1	ENSG00000269897	-0,606	1,09E-03	3,22E-02
CDK20	ENSG00000156345	0,409	1,09E-03	3,22E-02
ACTL10	ENSG00000288649	0,358	1,11E-03	3,23E-02
AKT3	ENSG00000275199	-0,276	1,11E-03	3,23E-02
BEST1	ENSG00000167995	3,000	1,10E-03	3,23E-02
ITPKC	ENSG00000086544	0,230	1,11E-03	3,23E-02
LRG1	ENSG00000171236	0,915	1,10E-03	3,23E-02
TNRC18	ENSG00000182095	-0,235	1,10E-03	3,23E-02
VPS8	ENSG00000156931	-0,319	1,10E-03	3,23E-02
PRRC2B	ENSG00000288701	-0,252	1,11E-03	3,23E-02
CHST6	ENSG00000183196	0,461	1,14E-03	3,30E-02
CLDN10	ENSG00000134873	-0,387	1,16E-03	3,36E-02
ELK3	ENSG00000111145	-0,267	1,16E-03	3,36E-02
IGFBP1	ENSG00000146678	-0,548	1,16E-03	3,36E-02
TXNIP	ENSG00000265972	-0,485	1,16E-03	3,36E-02
GLCE	ENSG00000138604	-0,244	1,17E-03	3,37E-02
PLCD3	ENSG00000161714	-0,285	1,17E-03	3,37E-02
CHKB	ENSG00000100288	0,197	1,19E-03	3,40E-02
POLD4	ENSG00000175482	0,346	1,20E-03	3,42E-02
FCF1	ENSG00000119616	-0,213	1,21E-03	3,43E-02
SP110	ENSG00000135899	0,241	1,20E-03	3,43E-02
LGALS3BP	ENSG00000108679	0,620	1,21E-03	3,43E-02
OAS3	ENSG00000111331	0,322	1,23E-03	3,49E-02
PLEKHA4	ENSG00000105559	0,272	1,24E-03	3,51E-02
ASPHD1	ENSG00000174939	0,680	1,26E-03	3,55E-02
ITPRIPL1	ENSG00000198885	0,456	1,26E-03	3,55E-02
S1PR2	ENSG00000267534	0,346	1,26E-03	3,55E-02
SIRT3	ENSG00000142082	0,207	1,26E-03	3,55E-02
UBA6	ENSG00000033178	-0,346	1,27E-03	3,56E-02
AMDHD2	ENSG00000162066	0,275	1,27E-03	3,56E-02
AGFG1	ENSG00000173744	-0,166	1,29E-03	3,60E-02
DEPDC1	ENSG00000024526	-0,581	1,31E-03	3,66E-02
NCEH1	ENSG00000144959	-0,174	1,32E-03	3,69E-02
STEAP1B	ENSG00000105889	-0,254	1,33E-03	3,71E-02
RMND5A	ENSG00000153561	-0,187	1,35E-03	3,74E-02

APPBP2	ENSG00000062725	-0,365	1,36E-03	3,76E-02
MALL	ENSG00000144063	-0,226	1,36E-03	3,76E-02
MAP3K5	ENSG00000197442	-0,280	1,37E-03	3,78E-02
CCS	ENSG00000173992	0,222	1,38E-03	3,81E-02
SELP	ENSG00000174175	-0,676	1,39E-03	3,81E-02
P4HA2	ENSG00000072682	0,217	1,40E-03	3,84E-02
NUDT18	ENSG00000275074	0,274	1,41E-03	3,86E-02
DOCK4	ENSG00000128512	-0,288	1,43E-03	3,91E-02
SERPINE1	ENSG00000106366	0,263	1,43E-03	3,91E-02
CDC20	ENSG00000117399	-0,358	1,44E-03	3,91E-02
LAMP2	ENSG00000005893	-0,141	1,44E-03	3,91E-02
SAMHD1	ENSG00000101347	-0,369	1,45E-03	3,92E-02
FANCG	ENSG00000221829	0,345	1,45E-03	3,94E-02
KYNU	ENSG00000115919	0,782	1,46E-03	3,95E-02
RSC1A1	ENSG00000215695	-0,388	1,47E-03	3,96E-02
SNX6	ENSG00000129515	-0,454	1,47E-03	3,97E-02
DIS3L	ENSG00000166938	-0,276	1,48E-03	3,98E-02
FLI1	ENSG00000151702	-0,212	1,48E-03	3,98E-02
SMG1P1	ENSG00000237296	-0,304	1,50E-03	4,00E-02
C6orf136	ENSG00000224120	0,223	1,51E-03	4,04E-02
ANKRD55	ENSG00000164512	0,227	1,53E-03	4,07E-02
NUSAP1	ENSG00000137804	-0,257	1,54E-03	4,10E-02
MCTP2	ENSG00000140563	0,553	1,55E-03	4,12E-02
SMIM10	ENSG00000184785	-0,426	1,56E-03	4,12E-02
IRF7	ENSG00000276561	0,301	1,56E-03	4,14E-02
FBXW4	ENSG00000107829	0,228	1,57E-03	4,15E-02
PDXK	ENSG00000160209	0,284	1,60E-03	4,22E-02
SIK1B	ENSG00000275993	0,287	1,62E-03	4,26E-02
ALDH3A2	ENSG00000072210	-0,186	1,62E-03	4,27E-02
MICAL1	ENSG00000135596	0,209	1,63E-03	4,27E-02
RNASEK	ENSG00000219200	0,259	1,63E-03	4,27E-02
POGLUT3	ENSG00000178202	-0,247	1,64E-03	4,29E-02
PIF1	ENSG00000140451	-0,318	1,69E-03	4,39E-02
SQLE	ENSG00000104549	-0,304	1,69E-03	4,39E-02
AP000295.1	NA	-0,791	1,72E-03	4,42E-02
ARHGAP29	ENSG00000137962	-0,385	1,70E-03	4,42E-02
HSD3B7	ENSG00000099377	0,300	1,71E-03	4,42E-02
KAT2B	ENSG00000114166	-0,353	1,72E-03	4,42E-02
LZTFL1	ENSG00000163818	-0,296	1,71E-03	4,42E-02
PARPBP	ENSG00000185480	-0,421	1,72E-03	4,42E-02
RRAS	ENSG00000126458	0,247	1,72E-03	4,42E-02
TAX1BP3	ENSG00000213977	0,221	1,70E-03	4,42E-02
YAP1	ENSG00000137693	-0,249	1,73E-03	4,42E-02
CFLAR	ENSG00000003402	0,156	1,74E-03	4,44E-02
ZBTB21	ENSG00000173276	-0,333	1,74E-03	4,44E-02
NPAS2	ENSG00000170485	-0,270	1,75E-03	4,45E-02
SPIN2B	ENSG00000186787	-0,244	1,75E-03	4,45E-02
ARHGAP4	ENSG00000089820	0,214	1,77E-03	4,48E-02
GPD1L	ENSG00000152642	-0,209	1,77E-03	4,48E-02
MEF2A	ENSG00000068305	-0,344	1,79E-03	4,52E-02
RGS5	ENSG00000232995	-0,291	1,80E-03	4,54E-02
YPEL3	ENSG00000090238	0,217	1,80E-03	4,54E-02
PRRX1	ENSG00000116132	1,003	1,81E-03	4,54E-02

YIPF1	ENSG00000058799	0,223	1,84E-03	4,62E-02
PIEZO2	ENSG00000154864	-0,312	1,85E-03	4,64E-02
IL1R1	ENSG00000115594	-0,282	1,86E-03	4,65E-02
SAMD8	ENSG00000156671	-0,225	1,86E-03	4,66E-02
NUDT4B	ENSG00000177144	-0,160	1,89E-03	4,73E-02
SULF1	ENSG00000137573	-0,415	1,90E-03	4,73E-02
ADGRF3	ENSG00000173567	2,100	1,91E-03	4,75E-02
PGM2	ENSG00000169299	-0,220	1,91E-03	4,75E-02
MOV10	ENSG00000155363	0,221	1,93E-03	4,78E-02
C1S	ENSG00000182326	1,036	1,95E-03	4,83E-02
FAM114A1	ENSG00000197712	-0,168	1,96E-03	4,83E-02
KPNA1	ENSG00000114030	-0,182	1,96E-03	4,83E-02
SCLT1	ENSG00000151466	-0,365	1,98E-03	4,88E-02
MARCHF2	ENSG00000099785	0,189	1,99E-03	4,89E-02
CMKLR1	ENSG00000174600	-1,002	2,00E-03	4,91E-02
DARS2	ENSG00000117593	-0,281	2,01E-03	4,92E-02
B3GAT3	ENSG00000149541	0,247	2,02E-03	4,92E-02
CCDC190	ENSG00000185860	-0,264	2,02E-03	4,92E-02
CD109	ENSG00000156535	-0,318	2,02E-03	4,92E-02
SBNO2	ENSG00000278788	0,304	2,03E-03	4,92E-02
STYXL1	ENSG00000127952	0,217	2,01E-03	4,92E-02
CYSTM1	ENSG00000120306	0,205	2,04E-03	4,95E-02
VPS26A	ENSG00000122958	-0,298	2,05E-03	4,96E-02
PTK2	ENSG00000169398	-0,273	2,06E-03	4,98E-02
PRKAR2B	ENSG00000284096	-0,307	2,07E-03	4,99E-02
RBPMS2	ENSG00000166831	0,311	2,07E-03	4,99E-02
RGS4	ENSG00000117152	-0,258	2,08E-03	5,00E-02
TAP2	ENSG00000225967	0,212	2,08E-03	5,00E-02

Supplementary Table S6: Differentially expressed genes upon LPS treatment of cells expressing APEX1(1-20). DGE calculated using the R package DESeq2 comparing sam-ples of cells transduced with the lentivirus expressing APEX1(1-20) and treated with active LPS versus treated with detoxified LPS. The L2FC (Log 2-fold change) states the average difference in gene expression between both treatments. Positive L2FC values denote upregulation by LPS treatment, negative values downregulation. Wald test from DESeq2 was used to calculate the significance of the change in the expression. The adjusted p-values take the number of tested genes into account, the threshold for the adjusted p-value was 0.05.

gene name	Ensembl gene ID	L2FC	p-value	adusted p-value
LAMC2	ENSG00000058085	2,107	1,12E-198	1,76E-194
SOD2	ENSG00000112096	1,108	1,25E-66	9,84E-63
EBI3	ENSG00000105246	2,271	1,43E-53	7,47E-50
CXCL6	ENSG00000124875	1,655	1,16E-49	4,57E-46
MMP10	ENSG00000166670	1,106	1,31E-46	4,12E-43
CCL2	ENSG00000108691	1,060	7,21E-45	1,88E-41
CTSS	ENSG00000163131	1,287	6,43E-41	1,44E-37
CFB	ENSG00000242335	1,565	1,12E-40	2,21E-37
UBD	ENSG00000226898	2,203	9,00E-40	1,57E-36
CTSK	ENSG00000143387	1,417	1,61E-36	2,53E-33
POU2F2	ENSG00000028277	1,240	1,29E-35	1,83E-32
LTB	ENSG00000223448	1,169	9,43E-27	1,23E-23
IGFBP3	ENSG00000146674	-0,776	2,00E-26	2,42E-23
IL32	ENSG00000008517	1,115	5,74E-26	6,43E-23
CNTNAP1	ENSG00000108797	0,597	1,69E-24	1,77E-21
IFI27	ENSG00000275214	0,640	2,88E-23	2,82E-20
PAPLN	ENSG00000100767	1,408	9,10E-23	8,39E-20
ANO9	ENSG00000185101	4,416	3,91E-20	3,41E-17
ISG20	ENSG00000172183	0,752	1,23E-19	1,02E-16
S100A3	ENSG00000188015	1,601	7,48E-19	5,87E-16
CXCL1	ENSG00000163739	0,959	1,42E-18	1,06E-15
CXCL5	ENSG00000163735	1,745	1,40E-17	9,96E-15
METTL7A	ENSG00000185432	-0,636	3,12E-16	2,13E-13
AC091951.4	NA	4,960	5,48E-16	3,58E-13
THSD4	ENSG00000187720	0,378	6,63E-16	4,16E-13
NEURL1B	ENSG00000214357	-0,822	3,17E-15	1,91E-12
CXCL8	ENSG00000169429	1,039	6,64E-15	3,86E-12
MAMDC2	ENSG00000278608	0,926	2,34E-14	1,31E-11
CR354443.1	NA	32,453	2,78E-14	1,50E-11
CCL15-CCL14	ENSG00000282521	-0,637	6,52E-14	3,30E-11
FCF1P2	ENSG00000228638	-23,428	6,46E-14	3,30E-11
MX1	ENSG00000157601	0,881	1,11E-13	5,45E-11
SLC7A2	ENSG00000003989	0,614	2,48E-13	1,18E-10
H3P6	ENSG00000235655	30,910	2,90E-13	1,34E-10
PLA2G4C	ENSG00000105499	0,669	3,43E-13	1,54E-10
TBC1D3K	ENSG00000275153	-30,254	6,41E-13	2,79E-10
HLA-B	ENSG00000206450	0,830	2,21E-12	9,37E-10
AL451062.4	NA	-29,257	3,51E-12	1,45E-09
LYPD6	ENSG00000187123	0,545	2,51E-11	1,01E-08
ITGB4	ENSG00000132470	-0,899	3,01E-11	1,18E-08
AKAP12	ENSG00000131016	0,392	3,13E-11	1,20E-08
ACBD7	ENSG00000176244	-0,963	3,63E-11	1,34E-08
PXDN	ENSG00000130508	0,668	3,67E-11	1,34E-08
LAMP3	ENSG00000078081	0,580	3,98E-11	1,42E-08
IL4I1	ENSG00000104951	0,582	1,45E-10	5,05E-08

LIPG	ENSG00000101670	0,605	1,48E-10	5,05E-08
CTHRC1	ENSG00000164932	0,586	1,60E-10	5,34E-08
ICAM1	ENSG00000090339	0,518	2,53E-10	8,27E-08
ALOX5AP	ENSG00000132965	1,792	4,57E-10	1,46E-07
CD69	ENSG00000110848	1,109	8,28E-10	2,60E-07
CYB5R2	ENSG00000166394	0,651	9,12E-10	2,80E-07
KLF2	ENSG00000127528	-0,494	1,42E-09	4,28E-07
MMP19	ENSG00000123342	0,700	1,82E-09	5,29E-07
SRP9P1	ENSG00000180581	25,646	1,82E-09	5,29E-07
CD34	ENSG00000174059	-0,395	1,98E-09	5,66E-07
PSMB9	ENSG00000243958	0,523	2,11E-09	5,90E-07
CXCL2	ENSG00000081041	0,820	3,17E-09	8,72E-07
IFI6	ENSG00000126709	0,493	4,18E-09	1,13E-06
KIT	ENSG00000157404	0,351	5,37E-09	1,43E-06
PPL	ENSG00000118898	-1,098	6,56E-09	1,72E-06
TFPI2	ENSG00000105825	0,607	6,91E-09	1,78E-06
C2CD4A	ENSG00000198535	0,816	1,09E-08	2,76E-06
AP005018.2	NA	-23,882	1,38E-08	3,43E-06
CCL14	ENSG00000277236	-0,599	1,43E-08	3,51E-06
F2RL1	ENSG00000164251	0,378	2,81E-08	6,78E-06
RNASE1	ENSG00000129538	-0,388	3,16E-08	7,52E-06
ALDH1A1	ENSG00000165092	-0,420	3,70E-08	8,66E-06
NID2	ENSG00000087303	0,901	4,00E-08	9,23E-06
NFKBIZ	ENSG00000144802	0,715	4,07E-08	9,25E-06
P2RX4	ENSG00000135124	0,313	6,22E-08	1,39E-05
SOX18	ENSG00000203883	-0,314	7,70E-08	1,70E-05
AQP1	ENSG00000240583	-1,437	9,16E-08	2,00E-05
CCNE1	ENSG00000105173	0,424	1,00E-07	2,16E-05
CXCL3	ENSG00000163734	0,498	1,33E-07	2,81E-05
CCL20	ENSG00000115009	0,878	1,41E-07	2,94E-05
ITGAV	ENSG00000138448	0,580	1,45E-07	2,99E-05
ACE	ENSG00000159640	-0,668	1,56E-07	3,18E-05
LYVE1	ENSG00000133800	-0,766	1,64E-07	3,29E-05
TNFRSF9	ENSG00000049249	1,044	2,28E-07	4,53E-05
STAP2	ENSG00000178078	0,406	2,37E-07	4,65E-05
PRICKLE1	ENSG00000139174	-0,462	2,88E-07	5,57E-05
OAS3	ENSG00000111331	0,509	3,18E-07	6,08E-05
ANGPT2	ENSG00000091879	0,361	4,34E-07	8,20E-05
BST1	ENSG00000109743	0,352	5,75E-07	1,07E-04
IL1A	ENSG00000115008	0,680	6,75E-07	1,25E-04
MEST	ENSG00000106484	-0,406	7,83E-07	1,43E-04
CCM2L	ENSG00000101331	-0,448	8,16E-07	1,47E-04
IFIT1	ENSG00000185745	0,962	9,22E-07	1,64E-04
CRACR2B	ENSG00000177685	-0,419	9,45E-07	1,65E-04
OAS1	ENSG00000089127	0,474	9,42E-07	1,65E-04
INHBB	ENSG00000163083	-0,814	9,82E-07	1,69E-04
PAPPA2	ENSG00000116183	1,562	9,88E-07	1,69E-04
TXNIP	ENSG00000265972	-0,730	1,01E-06	1,70E-04
GJA4	ENSG00000187513	-0,568	1,05E-06	1,75E-04
IGFBP4	ENSG00000141753	-0,294	1,18E-06	1,95E-04
TNFSF15	ENSG00000181634	0,311	1,38E-06	2,25E-04
ADAMTS18	ENSG00000140873	-0,514	1,55E-06	2,50E-04
FRY	ENSG00000073910	-0,217	1,61E-06	2,58E-04

PLCD3	ENSG00000161714	-0,419	1,81E-06	2,87E-04
IL7R	ENSG00000168685	0,419	1,89E-06	2,96E-04
TNC	ENSG00000041982	2,315	1,99E-06	3,09E-04
SYNJ2	ENSG00000078269	0,174	2,09E-06	3,21E-04
MSMP	ENSG00000215183	-0,386	3,03E-06	4,62E-04
GMFG	ENSG00000130755	0,286	3,12E-06	4,70E-04
OCIAD2	ENSG00000145247	0,388	3,39E-06	5,07E-04
TMEM120A	ENSG00000189077	0,384	3,56E-06	5,27E-04
CSF2	ENSG00000164400	1,186	3,78E-06	5,54E-04
CREB5	ENSG00000146592	0,326	4,06E-06	5,89E-04
EPSTI1	ENSG00000133106	0,947	4,15E-06	5,93E-04
KIF20A	ENSG00000112984	-0,438	4,16E-06	5,93E-04
PRIM1	ENSG00000198056	0,506	4,50E-06	6,36E-04
TMEM184A	ENSG00000164855	1,991	4,78E-06	6,69E-04
OAS2	ENSG00000111335	0,745	5,53E-06	7,67E-04
CAV1	ENSG00000105974	-0,277	7,04E-06	9,64E-04
NRROS	ENSG00000174004	-0,303	7,06E-06	9,64E-04
TNIP3	ENSG00000050730	1,239	7,28E-06	9,85E-04
ADAMTS15	ENSG00000166106	-1,383	7,59E-06	1,02E-03
TAPBP	ENSG00000206281	0,364	8,63E-06	1,15E-03
ABCG2	ENSG00000118777	-0,578	9,42E-06	1,24E-03
NLRC3	ENSG00000167984	0,376	9,48E-06	1,24E-03
LRRC75A	ENSG00000181350	-0,393	1,00E-05	1,30E-03
PRSS12	ENSG00000164099	0,973	1,02E-05	1,32E-03
IFI44L	ENSG00000137959	0,935	1,18E-05	1,51E-03
EGLN3	ENSG00000129521	-1,171	1,28E-05	1,62E-03
TSPAN11	ENSG00000110900	-0,436	1,33E-05	1,67E-03
QPCT	ENSG00000115828	0,564	1,38E-05	1,72E-03
TNFRSF4	ENSG00000186827	0,805	1,41E-05	1,75E-03
COL1A2	ENSG00000164692	-0,415	1,53E-05	1,87E-03
CCNB2	ENSG00000157456	-0,387	1,64E-05	1,98E-03
EXO1	ENSG00000174371	0,511	1,64E-05	1,98E-03
CFLAR	ENSG00000003402	0,213	1,90E-05	2,27E-03
ZNF365	ENSG00000138311	0,547	2,07E-05	2,47E-03
ALDH1A2	ENSG00000128918	-0,324	2,29E-05	2,68E-03
GALNT18	ENSG00000110328	0,563	2,28E-05	2,68E-03
APLN	ENSG00000171388	-0,484	2,33E-05	2,71E-03
HMGCS1	ENSG00000112972	-0,321	2,41E-05	2,78E-03
ARHGDIG	ENSG00000242173	0,442	2,47E-05	2,83E-03
ZC3H12A	ENSG00000163874	0,454	2,53E-05	2,87E-03
ZNF467	ENSG00000181444	-0,453	3,02E-05	3,40E-03
SELENOT	ENSG00000198843	0,254	3,21E-05	3,59E-03
ACAT2	ENSG00000120437	-0,283	3,27E-05	3,61E-03
GPX3	ENSG00000211445	-0,573	3,27E-05	3,61E-03
GGT5	ENSG00000099998	-0,353	3,35E-05	3,66E-03
HLA-A	ENSG00000227715	0,289	3,36E-05	3,66E-03
SEMA7A	ENSG00000288455	0,486	3,81E-05	4,13E-03
YIPF5	ENSG00000145817	0,269	3,90E-05	4,19E-03
ASS1	ENSG00000130707	-0,446	4,11E-05	4,39E-03
MGARP	ENSG00000137463	-0,451	4,35E-05	4,61E-03
FP565260.3	NA	0,386	4,38E-05	4,61E-03
MALL	ENSG00000144063	-0,288	4,47E-05	4,67E-03
MRAP2	ENSG00000135324	-0,384	4,63E-05	4,81E-03

IL1RL1	ENSG00000115602	0,254	4,78E-05	4,90E-03
RAMP2	ENSG00000131477	-0,465	4,75E-05	4,90E-03
CD44	ENSG00000026508	0,503	5,20E-05	5,30E-03
LRTOMT	ENSG00000184154	-0,402	5,45E-05	5,51E-03
AC005520.3	NA	-1,051	5,60E-05	5,63E-03
CSF3	ENSG00000108342	1,064	5,92E-05	5,91E-03
FBXO32	ENSG00000156804	0,351	6,03E-05	5,99E-03
DHH	ENSG00000139549	-0,365	6,08E-05	5,99E-03
RALB	ENSG00000144118	-0,201	6,25E-05	6,13E-03
HPSE	ENSG00000173083	0,245	6,31E-05	6,15E-03
LGALS3BP	ENSG00000108679	0,760	6,40E-05	6,19E-03
TUSC3	ENSG00000104723	0,294	6,43E-05	6,19E-03
SH3BP1	ENSG00000100092	0,715	6,69E-05	6,40E-03
SMIM3	ENSG00000256235	-0,424	6,75E-05	6,42E-03
NPTN	ENSG00000156642	0,193	6,88E-05	6,51E-03
ABLIM2	ENSG00000163995	-1,741	8,11E-05	7,58E-03
MYRIP	ENSG00000170011	-0,301	8,15E-05	7,58E-03
TCF15	ENSG00000125878	-0,467	8,17E-05	7,58E-03
C4B	ENSG00000224639	-1,377	8,74E-05	7,99E-03
CASP1	ENSG00000137752	0,501	8,76E-05	7,99E-03
KIF12	ENSG00000136883	0,542	8,73E-05	7,99E-03
SERPINA3	ENSG00000196136	1,848	8,87E-05	8,05E-03
COLGALT1	ENSG00000130309	0,367	9,66E-05	8,71E-03
AFF3	ENSG00000144218	1,033	1,05E-04	9,24E-03
EIF3CL	ENSG00000205609	-0,720	1,04E-04	9,24E-03
ERMP1	ENSG00000099219	-0,243	1,04E-04	9,24E-03
HLA-H	ENSG00000231904	0,357	1,05E-04	9,24E-03
SPACA6	ENSG00000182310	-0,405	1,04E-04	9,24E-03
CCNB1	ENSG00000134057	-0,396	1,06E-04	9,27E-03
MYZAP	ENSG00000263155	-0,356	1,19E-04	1,03E-02
C20orf204	ENSG00000196421	-0,498	1,21E-04	1,04E-02
CGNL1	ENSG00000128849	-0,203	1,21E-04	1,04E-02
SLC6A15	ENSG00000072041	0,655	1,22E-04	1,04E-02
DHRS3	ENSG00000162496	0,284	1,24E-04	1,05E-02
OASL	ENSG00000135114	1,044	1,24E-04	1,05E-02
DNAH5	ENSG00000039139	0,437	1,26E-04	1,05E-02
FBLN2	ENSG00000163520	-0,677	1,26E-04	1,05E-02
AOX1	ENSG00000138356	0,620	1,39E-04	1,15E-02
ICOSLG	ENSG00000160223	0,377	1,40E-04	1,15E-02
LHX6	ENSG00000106852	-0,471	1,41E-04	1,15E-02
SLIT3	ENSG00000184347	0,552	1,42E-04	1,16E-02
AC019117.4	NA	8,471	1,44E-04	1,17E-02
MAP3K6	ENSG00000142733	0,299	1,49E-04	1,20E-02
ABI3BP	ENSG00000154175	0,365	1,52E-04	1,22E-02
KYNU	ENSG00000115919	0,927	1,53E-04	1,22E-02
SAPCD2	ENSG00000186193	-0,366	1,52E-04	1,22E-02
MTMR7	ENSG00000003987	0,923	1,53E-04	1,22E-02
GIMAP1	ENSG00000213203	-0,265	1,60E-04	1,26E-02
NQO1	ENSG00000181019	-0,281	1,60E-04	1,26E-02
CASKIN1	ENSG00000167971	-2,314	1,67E-04	1,30E-02
CA4	ENSG00000167434	-1,172	1,76E-04	1,37E-02
LY75-CD302	ENSG00000248672	0,547	1,79E-04	1,38E-02
AC244260.1	NA	8,622	1,92E-04	1,48E-02

CDH4	ENSG00000280641	-0,476	1,93E-04	1,48E-02
GALNT12	ENSG00000119514	0,363	1,95E-04	1,49E-02
PCK2	ENSG00000285241	0,283	2,15E-04	1,63E-02
ABCC2	ENSG00000023839	-0,663	2,17E-04	1,63E-02
CAMSAP3	ENSG00000076826	-0,935	2,17E-04	1,63E-02
GUCY1A1	ENSG00000164116	-0,911	2,21E-04	1,65E-02
MAP3K14	ENSG00000282637	-0,384	2,28E-04	1,70E-02
ALDH1L2	ENSG00000136010	0,311	2,30E-04	1,70E-02
IFI44	ENSG00000137965	0,499	2,32E-04	1,71E-02
BCAM	ENSG00000187244	-0,275	2,37E-04	1,74E-02
TCF7	ENSG00000081059	0,391	2,44E-04	1,78E-02
SCNN1B	ENSG00000168447	-2,117	2,45E-04	1,78E-02
CHRNA1	ENSG00000138435	0,426	2,48E-04	1,79E-02
PLAAT4	ENSG00000133321	0,548	2,56E-04	1,84E-02
CYP1A1	ENSG00000140465	-0,508	2,66E-04	1,91E-02
PRCP	ENSG00000137509	0,175	2,71E-04	1,93E-02
NOS3	ENSG00000164867	-0,330	2,80E-04	1,99E-02
CLEC14A	ENSG00000176435	-0,190	2,82E-04	1,99E-02
IFI35	ENSG00000068079	0,344	2,86E-04	2,01E-02
PTGS2	ENSG00000073756	0,572	2,92E-04	2,05E-02
POSTN	ENSG00000133110	-0,539	3,01E-04	2,10E-02
DUXAP9	ENSG00000225210	0,284	3,07E-04	2,13E-02
MYPN	ENSG00000138347	0,620	3,15E-04	2,17E-02
PIR	ENSG00000087842	-0,566	3,27E-04	2,25E-02
AK4	ENSG00000162433	0,170	3,35E-04	2,29E-02
IFT122	ENSG00000163913	-0,270	3,36E-04	2,29E-02
P2RY6	ENSG00000171631	1,128	3,53E-04	2,40E-02
DPYSL3	ENSG00000113657	0,257	3,60E-04	2,43E-02
ATP2B4	ENSG00000058668	-0,175	3,69E-04	2,48E-02
GK	ENSG00000198814	0,285	3,79E-04	2,52E-02
SELENOM	ENSG00000198832	0,345	3,78E-04	2,52E-02
ZNF219	ENSG00000165804	-0,385	3,80E-04	2,52E-02
PIEZO2	ENSG00000154864	-0,355	3,88E-04	2,57E-02
STC1	ENSG00000159167	-0,462	3,91E-04	2,58E-02
APOD	ENSG00000189058	0,347	4,06E-04	2,64E-02
ATAD2	ENSG00000156802	0,366	4,10E-04	2,64E-02
BUB1	ENSG00000169679	-0,310	4,11E-04	2,64E-02
GPNMB	ENSG00000136235	-1,050	4,09E-04	2,64E-02
NOS1	ENSG00000089250	-2,260	4,09E-04	2,64E-02
PCDH1	ENSG00000156453	0,264	4,06E-04	2,64E-02
DLL1	ENSG00000275555	-0,370	4,21E-04	2,69E-02
ENOSF1	ENSG00000132199	-0,301	4,25E-04	2,71E-02
TMOD1	ENSG00000136842	0,445	4,30E-04	2,72E-02
TSPAN8	ENSG00000127324	-0,646	4,30E-04	2,72E-02
CLEC3B	ENSG00000163815	-1,195	4,35E-04	2,74E-02
SLCO2A1	ENSG00000174640	-0,674	4,36E-04	2,74E-02
PLA2G5	ENSG00000127472	1,414	4,50E-04	2,80E-02
RASSF10	ENSG00000189431	-1,470	4,49E-04	2,80E-02
AMPD3	ENSG00000133805	0,780	4,55E-04	2,81E-02
LRP12	ENSG00000147650	0,252	4,54E-04	2,81E-02
CHAF1A	ENSG00000167670	0,466	4,61E-04	2,83E-02
PRRT2	ENSG00000167371	2,229	4,61E-04	2,83E-02
HSPB6	ENSG00000004776	-0,351	4,65E-04	2,83E-02

WNT9A	ENSG00000143816	-0,439	4,66E-04	2,83E-02
CACHD1	ENSG00000158966	0,277	4,80E-04	2,91E-02
GSTM2	ENSG00000213366	-0,342	4,84E-04	2,92E-02
CD36	ENSG00000135218	-0,760	4,87E-04	2,93E-02
TNFSF18	ENSG00000120337	-0,366	5,05E-04	3,02E-02
CST1	ENSG00000170373	0,610	5,07E-04	3,02E-02
KCNN4	ENSG00000104783	-0,792	5,12E-04	3,04E-02
SHE	ENSG00000169291	-0,219	5,15E-04	3,05E-02
DUSP4	ENSG00000120875	-0,261	5,23E-04	3,09E-02
CENPBD1	ENSG00000177946	0,199	5,36E-04	3,14E-02
TM7SF2	ENSG00000149809	-0,335	5,35E-04	3,14E-02
HSPB8	ENSG00000152137	0,334	5,44E-04	3,18E-02
IGFBP1	ENSG00000146678	-0,582	5,65E-04	3,27E-02
UBE2L6	ENSG00000156587	0,205	5,64E-04	3,27E-02
SYT7	ENSG00000011347	-0,953	5,70E-04	3,29E-02
SLC15A3	ENSG00000110446	0,327	5,73E-04	3,29E-02
ZFPM2	ENSG00000169946	0,369	5,92E-04	3,39E-02
CDC20	ENSG00000117399	-0,386	6,03E-04	3,44E-02
ANKRD44	ENSG00000065413	-0,767	6,07E-04	3,45E-02
NTSR1	ENSG00000101188	-0,347	6,10E-04	3,46E-02
DNAH11	ENSG00000105877	0,366	6,21E-04	3,50E-02
CLSPN	ENSG00000092853	0,378	6,31E-04	3,55E-02
FAM221A	ENSG00000188732	-0,310	6,40E-04	3,55E-02
PIK3R3	ENSG00000117461	-0,311	6,35E-04	3,55E-02
PLSCR1	ENSG00000188313	0,208	6,38E-04	3,55E-02
RTN4RL1	ENSG00000185924	-2,401	6,39E-04	3,55E-02
BDKRB2	ENSG00000168398	0,651	6,48E-04	3,55E-02
CDC25B	ENSG00000101224	-0,240	6,46E-04	3,55E-02
MOBP	ENSG00000168314	0,795	6,44E-04	3,55E-02
NFIA	ENSG00000162599	-0,403	6,63E-04	3,63E-02
GIMAP8	ENSG00000171115	-0,227	6,76E-04	3,68E-02
PARD6A	ENSG00000102981	-0,362	6,88E-04	3,74E-02
ADGRF3	ENSG00000173567	2,256	7,00E-04	3,78E-02
PSMB8	ENSG00000230669	0,220	7,14E-04	3,85E-02
CCL23	ENSG00000276114	-0,826	7,24E-04	3,88E-02
RASGRF2	ENSG00000113319	0,347	7,24E-04	3,88E-02
C1orf115	ENSG00000162817	-0,180	7,31E-04	3,90E-02
NUAK1	ENSG00000074590	0,442	7,38E-04	3,93E-02
AUNIP	ENSG00000127423	0,412	7,58E-04	4,00E-02
FSTL3	ENSG00000070404	0,289	7,58E-04	4,00E-02
ARSK	ENSG00000164291	0,211	7,70E-04	4,04E-02
MXD3	ENSG00000213347	-0,387	7,69E-04	4,04E-02
CLEC1A	ENSG00000150048	0,182	7,77E-04	4,06E-02
HTR2B	ENSG00000135914	-0,366	8,16E-04	4,25E-02
BTD	ENSG00000169814	-0,185	8,29E-04	4,29E-02
TCN2	ENSG00000185339	-0,295	8,26E-04	4,29E-02
TSPAN5	ENSG00000168785	-0,178	8,41E-04	4,34E-02
CIT	ENSG00000122966	-0,302	8,70E-04	4,48E-02
ZDHHC13	ENSG00000177054	-0,211	8,97E-04	4,60E-02
ACKR4	ENSG00000129048	-0,454	9,16E-04	4,63E-02
ARL14EPL	ENSG00000268223	-0,995	9,21E-04	4,63E-02
MATN2	ENSG00000132561	-0,529	9,21E-04	4,63E-02
PDE1C	ENSG00000154678	0,280	9,17E-04	4,63E-02

RGS2	ENSG00000116741	0,398	9,15E-04	4,63E-02
SULF1	ENSG00000137573	-0,443	9,12E-04	4,63E-02
CYP51A1	ENSG00000001630	-0,193	9,28E-04	4,65E-02
ARHGEF26	ENSG00000277101	0,351	9,35E-04	4,67E-02
TUBB2B	ENSG00000137285	0,445	9,73E-04	4,84E-02
BTG2	ENSG00000159388	-0,190	9,94E-04	4,93E-02

Supplementary Table S7: Genes upregulated by LPS exclusively in cells that do not express APEX1(1-20). To identify genes upregulated by LPS specifically in cells transduced with the empty virus, but not in cells expressing moderate levels of APEX1(1- 20), the results of the DGE analysis of both cells populations after treatment with detoxified (con) or active LPS (LPS) were combined. The L2FC (Log 2-fold change) states the average difference in gene expression between both treatments, positive values denote upregulation by LPS treatment. Wald test from DESeq2 was used to calculate the significance of the change in the expression. The adjusted p-values take the number of tested genes into account, the threshold for the adjusted p-value was 0.05. Mean expression levels per sample group are stated in transcripts per million (TPM), calculated during quasi-mapping with the tool salmon. The list is sorted by gene expression.

gene name	L2FC	adjusted p-value	mean TPM empty virus con	mean TPM empty virus LPS	mean TPM APEX1(1-20) con	mean TPM APEX1(1-20) LPS
FTH1	0,268	4,30E-03	4639,36	5565,16	4723,13	5429,98
SERPINE1	0,263	3,91E-02	2753,76	3306,84	2860,91	3352,05
SAT1	0,334	2,28E-02	372,10	468,58	420,02	491,31
RNASEK	0,259	4,27E-02	302,79	362,46	319,66	347,36
GDF15	0,428	4,16E-03	181,22	243,01	186,96	225,56
TAX1BP3	0,221	4,42E-02	166,16	193,15	174,50	184,39
COTL1	0,280	6,99E-03	165,55	200,62	173,08	192,33
COL4A2	0,432	2,82E-02	148,83	198,87	156,04	201,24
PSME1	0,266	4,11E-03	147,10	176,17	153,10	168,61
PSME2	0,443	8,81E-05	134,36	181,67	140,36	171,61
RRAS	0,247	4,42E-02	128,19	152,15	131,01	143,27
HLA-C	0,313	8,89E-03	120,68	149,68	127,87	149,23
MT1L	0,355	1,88E-02	119,01	151,73	116,61	144,39
IER3	0,270	7,20E-03	100,45	120,93	107,70	115,41
ATP5F1D	0,341	1,18E-02	92,26	116,67	100,23	97,38
MFAP2	0,276	5,43E-03	70,80	85,75	75,64	78,59
CYSTM1	0,205	4,95E-02	68,11	78,37	68,82	69,97
SPHK1	0,285	2,16E-02	64,57	78,63	65,73	71,53
PDXK	0,284	4,22E-02	63,89	77,58	69,04	72,86
STYXL1	0,217	4,92E-02	55,70	64,54	53,68	57,32
BPGM	0,259	1,57E-02	47,34	56,61	48,03	56,46
PLCG1	0,243	5,56E-04	46,66	55,06	50,10	50,59
NFKBIA	0,272	5,05E-03	44,13	53,19	48,70	53,45
P4HA2	0,217	3,84E-02	41,12	47,65	40,49	44,88
ISG15	0,345	1,23E-02	40,32	51,21	43,32	50,22
SERPINB9	0,176	2,04E-02	40,12	45,26	43,72	46,50
DPP3	0,281	5,75E-03	39,09	47,59	39,50	45,15
CXCR4	0,237	1,68E-02	37,57	44,12	36,06	41,30
TSPAN13	0,390	8,14E-04	36,14	47,46	38,66	43,40
BST2	0,315	7,15E-04	35,86	44,46	37,59	42,84
SIPA1	0,279	3,13E-02	32,32	39,17	34,52	35,80
TMEM132A	0,351	6,18E-03	31,27	39,93	34,67	35,67
POLD4	0,346	3,42E-02	28,60	36,48	29,78	31,91
UPP1	0,232	1,96E-02	28,15	32,94	27,23	31,38
GMPPA	0,312	1,03E-02	28,14	34,92	28,84	32,19
PSMB10	0,328	2,68E-03	27,59	34,55	28,71	32,02
YPEL3	0,217	4,54E-02	26,38	30,64	29,57	28,61
GSDMD	0,274	6,99E-03	25,96	31,39	27,55	29,02
FHL3	0,276	3,00E-02	25,68	31,13	27,18	29,16
PLTP	0,355	5,67E-03	25,47	32,76	27,27	31,30
MARCHF2	0,189	4,89E-02	23,89	27,20	26,73	24,84
TMEM54	0,266	8,15E-03	23,67	28,40	25,16	25,07
NUDT22	0,284	3,13E-02	22,84	27,79	24,16	24,80
TNFRSF21	0,172	3,10E-02	22,78	25,62	24,02	26,02
UBR4	0,376	1,12E-02	22,72	29,57	22,32	25,97

ANKH	0,290	8,12E-03	22,47	27,49	22,50	24,26
GRASP	0,197	5,05E-03	22,21	25,48	23,13	23,42
AGRN	0,368	1,28E-02	21,33	27,78	21,89	26,02
MOV10	0,221	4,78E-02	20,49	23,73	21,01	20,55
FBLN5	0,404	1,15E-02	19,17	25,33	21,56	26,50
TSSC4	0,419	1,28E-02	18,98	25,48	19,43	19,63
TCIRG1	0,271	1,43E-02	18,05	21,85	19,29	20,30
EML2	0,311	1,35E-02	17,59	21,82	19,57	18,49
SURF1	0,234	9,64E-03	17,51	20,53	18,53	18,21
RSAD1	0,197	1,18E-02	16,94	19,36	17,55	18,60
CCS	0,222	3,81E-02	16,90	19,69	17,92	17,70
GBGT1	0,343	1,72E-02	16,83	21,36	17,33	19,51
SLC35C2	0,269	1,84E-02	16,83	20,30	17,60	18,66
CHKB	0,197	3,40E-02	16,68	19,04	17,40	17,25
LIG1	0,274	1,63E-02	16,52	19,88	15,96	18,60
SDC4	0,352	6,93E-04	16,38	20,79	17,77	19,54
STARD10	0,569	4,79E-07	16,35	24,40	18,98	22,60
SLC38A5	0,391	1,91E-02	16,17	21,13	18,27	19,16
MAP1LC3A	0,328	1,88E-02	15,91	19,99	17,73	18,79
LRFN4	0,375	9,94E-04	15,46	19,96	17,12	17,34
APOL1	0,388	1,24E-06	14,89	19,46	17,09	18,97
CAMTA2	0,290	3,88E-03	14,70	17,96	13,71	15,09
CRTAC1	0,499	2,49E-05	14,63	20,56	15,89	19,72
YIPF1	0,223	4,62E-02	14,38	16,72	14,35	15,65
MICAL1	0,209	4,27E-02	14,02	16,23	14,04	15,16
TNFRSF6B	0,338	4,52E-03	13,87	17,58	14,85	15,80
JUNB	0,259	1,57E-02	13,62	16,41	14,14	15,42
SBNO2	0,304	4,92E-02	13,55	16,80	14,23	16,14
NFATC4	0,371	1,94E-02	13,55	17,33	16,04	14,90
C6orf136	0,223	4,04E-02	13,28	15,48	12,90	13,51
POLD1	0,320	2,33E-02	12,27	15,26	12,21	14,22
B3GAT3	0,247	4,92E-02	12,23	14,49	12,50	13,71
GALT	0,230	2,51E-02	12,15	14,22	13,09	13,54
PTK7	0,269	4,49E-03	12,13	14,54	12,75	13,40
CCDC28B	0,254	2,57E-02	11,16	13,29	11,61	12,56
TAP2	0,212	5,00E-02	10,59	12,21	10,79	11,33
TNFRSF14	0,388	1,03E-03	10,32	13,55	11,52	13,07
IFITM1	0,366	2,77E-03	10,28	13,27	11,68	13,46
TMC6	0,276	2,09E-02	9,99	12,12	10,48	11,78
CBR3	0,357	1,62E-03	9,82	12,53	10,15	11,75
FBXW4	0,228	4,15E-02	9,73	11,36	10,21	10,78
FANCG	0,345	3,94E-02	9,48	12,03	9,66	10,17
SLC7A7	0,298	1,68E-02	9,01	11,09	9,71	10,53
ITPKC	0,230	3,23E-02	8,88	10,40	9,02	9,99
MFSD3	0,235	3,15E-02	8,42	9,89	8,93	8,74
SP110	0,241	3,43E-02	8,34	9,85	8,64	9,84
IRF9	0,498	1,26E-03	8,34	11,68	8,74	10,84
SIRT3	0,207	3,55E-02	8,30	9,56	8,63	8,25
UBA7	0,446	6,02E-06	7,69	10,51	8,69	10,17
TREX1	0,275	2,37E-02	7,35	8,84	7,15	8,25
IL3RA	0,238	2,67E-02	7,32	8,63	8,49	8,64
PARP10	0,319	1,05E-02	7,31	9,12	7,17	8,69
SLC27A3	0,380	1,86E-02	7,02	9,15	7,23	8,06

ULBP2	0,338	1,95E-03	6,99	8,81	7,68	8,43
C19orf47	0,357	2,99E-02	6,93	8,83	7,20	8,14
ARHGAP4	0,214	4,48E-02	6,92	8,02	7,26	6,77
RELB	0,353	9,55E-03	6,79	8,68	7,33	7,87
MS4A6A	0,466	5,66E-03	6,75	9,31	6,92	8,42
LAMB3	0,446	2,35E-03	6,66	9,08	6,82	7,85
SOCS3	0,302	1,15E-02	6,29	7,77	6,34	7,05
AMDHD2	0,275	3,56E-02	6,18	7,50	6,51	7,01
ARSA	0,292	1,15E-02	6,12	7,50	6,64	7,17
TCEAL7	0,328	5,88E-03	6,06	7,61	7,03	7,48
PMM1	0,341	1,30E-02	5,47	6,91	5,80	6,62
RPS6KL1	0,352	4,35E-03	5,18	6,57	6,25	6,57
APOBEC3G	0,249	2,89E-02	5,17	6,13	5,50	6,00
SAMD14	0,369	6,93E-04	5,09	6,59	5,72	6,44
E2F1	0,419	2,20E-02	5,08	6,74	5,14	6,70
RND1	0,401	6,28E-03	4,89	6,53	5,96	7,31
MTMR11	0,364	1,27E-03	4,50	5,80	4,61	5,11
PLCG2	1,263	8,65E-05	4,47	10,93	6,94	9,00
GFPT2	0,367	1,51E-02	4,46	5,74	4,86	6,11
PAQR7	0,277	1,42E-04	4,36	5,28	4,58	4,75
BLOC1S3	0,447	2,87E-02	4,25	5,83	5,33	4,58
NUDT18	0,274	3,86E-02	4,17	5,03	4,43	4,75
RASD1	0,316	7,60E-03	4,10	5,11	4,04	4,80
ANKRD55	0,227	4,07E-02	3,96	4,62	4,03	4,54
CERS1	0,381	4,90E-04	3,71	4,83	3,84	4,15
PLEKHA4	0,272	3,51E-02	3,61	4,37	3,79	4,16
TAPBPL	0,812	1,61E-04	3,35	5,70	4,36	4,51
SLC1A4	0,234	8,33E-03	3,22	3,78	3,42	3,70
CPLANE2	0,390	3,83E-03	3,18	4,17	3,23	3,57
SEMA3G	0,417	2,76E-04	3,17	4,24	3,57	4,37
CFAP410	0,304	3,05E-02	3,16	3,91	3,60	3,41
GBP4	0,310	9,61E-03	3,06	3,80	3,21	3,55
ACBD4	0,278	1,93E-02	2,93	3,55	3,31	3,18
CEBPD	0,785	1,63E-06	2,89	5,00	3,62	4,84
ACSS1	0,279	1,11E-04	2,85	3,45	2,97	3,29
RASA4	0,440	5,93E-04	2,80	3,81	3,00	3,68
IL27RA	0,362	1,14E-03	2,67	3,43	2,87	3,38
AMPH	0,488	6,61E-03	2,65	3,70	3,11	4,11
IRF7	0,301	4,14E-02	2,59	3,20	2,64	3,21
ASPHD2	0,209	3,21E-02	2,57	2,97	2,85	3,03
DENND2B	0,984	5,79E-06	2,56	4,92	3,05	3,49
PIK3CD	0,296	2,16E-02	2,54	3,14	2,75	3,18
SIK1B	0,287	4,26E-02	2,53	3,07	2,62	2,94
TMEM121	0,381	2,69E-03	2,44	3,17	2,82	2,99
C2CD4B	0,558	2,54E-02	2,43	3,57	2,65	3,46
TYMP	0,669	3,16E-02	2,16	3,43	3,14	3,38
FAHD2CP	0,381	2,59E-03	2,10	2,73	2,39	2,42
DHX58	0,330	2,91E-02	2,01	2,53	2,18	2,56
C11orf96	0,453	5,30E-04	1,98	2,72	2,22	2,34
VWA1	0,476	8,05E-07	1,95	2,71	2,18	2,54
RBPMS2	0,311	4,99E-02	1,84	2,28	1,97	2,08
SYNGR3	0,367	5,43E-03	1,82	2,36	2,02	2,27
ATOH8	0,364	2,02E-02	1,80	2,32	1,95	2,21

ARHGEF19	0,356	2,64E-04	1,59	2,04	1,71	1,88
TGFBR3L	0,467	6,18E-03	1,51	2,09	1,60	1,69
LRRC3	0,273	3,05E-02	1,31	1,58	1,35	1,52
RASA4B	0,567	1,42E-04	1,29	1,90	1,43	1,79
ZBTB42	0,284	1,87E-02	1,28	1,55	1,37	1,51
HSD3B7	0,300	4,42E-02	1,21	1,50	1,34	1,44
ITPRIPL1	0,456	3,55E-02	1,08	1,48	1,41	1,49
B3GALT4	0,398	5,43E-03	0,99	1,30	1,03	1,16
CX3CL1	0,553	1,96E-03	0,93	1,37	1,30	1,38
FMNL1	0,395	1,74E-02	0,93	1,23	1,10	1,15
AC005363.1	6,479	3,18E-02	0,89	3,89	2,74	2,46
ASPHD1	0,680	3,55E-02	0,87	1,41	1,44	1,44
CPAMD8	0,499	1,88E-02	0,77	1,10	0,82	0,96
FXYD6	0,656	9,95E-04	0,75	1,19	0,96	1,14
ODF3B	0,753	2,04E-02	0,74	1,25	0,82	1,21
MROH6	0,678	1,94E-02	0,69	1,10	1,10	0,90
ADM2	0,487	2,59E-02	0,65	0,91	0,65	0,81
FXYD2	0,734	1,43E-02	0,63	1,04	0,67	0,79
BATF3	0,662	2,60E-02	0,63	0,99	0,73	0,85
SLC22A31	0,695	3,88E-03	0,58	0,93	0,79	0,87
MELTF	0,373	3,00E-02	0,55	0,71	0,61	0,69
PLEKHN1	0,492	1,74E-02	0,54	0,76	0,52	0,70
TPPP3	0,496	2,61E-02	0,54	0,75	0,51	0,70
CDK20	0,409	3,22E-02	0,53	0,71	0,58	0,63
ACTL10	0,358	3,23E-02	0,50	0,64	0,54	0,56
HOXB9	0,395	1,14E-02	0,49	0,65	0,48	0,58
ADGRB2	0,324	2,01E-02	0,47	0,59	0,50	0,57
MCTP2	0,553	4,12E-02	0,44	0,64	0,58	0,53
ST6GALNAC2	0,756	1,60E-02	0,37	0,63	0,46	0,53
CDH15	0,509	1,57E-02	0,36	0,52	0,40	0,51
EFNA2	0,374	2,30E-02	0,34	0,44	0,39	0,39
JAK3	0,743	1,86E-05	0,33	0,56	0,40	0,55
C17orf107	0,459	4,81E-03	0,32	0,43	0,35	0,44
S1PR2	0,346	3,55E-02	0,29	0,37	0,33	0,37
NCKAP5	0,847	1,35E-02	0,27	0,49	0,33	0,50
NMNAT2	0,987	1,77E-02	0,21	0,41	0,27	0,35
PRRX1	1,003	4,54E-02	0,18	0,35	0,25	0,39
GXYLT2	1,226	2,85E-04	0,16	0,38	0,22	0,39
CLDN14	0,877	3,35E-03	0,15	0,28	0,20	0,25
CHST6	0,461	3,30E-02	0,15	0,21	0,15	0,21
SNX22	0,983	6,88E-03	0,14	0,29	0,16	0,18
ACHE	0,801	4,26E-03	0,13	0,23	0,14	0,22
IL11	0,997	8,34E-03	0,10	0,21	0,12	0,18
PTGES3L	1,020	1,79E-02	0,10	0,20	0,15	0,16
PLA1A	1,472	1,66E-06	0,10	0,27	0,14	0,24
C1S	1,036	4,83E-02	0,09	0,17	0,15	0,20
BEST1	3,000	3,23E-02	0,08	0,53	0,13	0,17
THBS2	0,836	1,44E-02	0,08	0,14	0,13	0,14
HAS2	1,050	6,88E-03	0,07	0,15	0,08	0,14
LRG1	0,915	3,23E-02	0,07	0,13	0,11	0,13
MAP6	0,992	5,88E-03	0,07	0,14	0,08	0,09
RAB7B	1,191	6,46E-03	0,05	0,12	0,08	0,10
RPL23AP87	2,313	3,21E-02	0,04	0,13	0,10	0,09

ADAM12	1,044	5,51E-03	0,04	0,08	0,04	0,07
MX2	2,548	8,60E-07	0,02	0,13	0,08	0,13
AL358472.7	20,830	1,46E-05	0,00	0,08	0,15	0,14

Supplementary Table S8: Genes downregulated by LPS exclusively in cells that do not express APEX1(1-20). To identify genes downregulated by LPS specifically in cells transduced with the empty virus, but not in cells expressing moderate levels of APEX1(1-20), the results of the DGE analysis of both cells populations after treatment with detoxified (con) or active LPS (LPS) were combined. The L2FC (Log 2-fold change) states the average difference in gene expression between both treatments, negative values denote downregulation by LPS treatment. Wald test from DESeq2 was used to calculate the significance of the change in the expression. The adjusted p-values take the number of tested genes into account, the threshold for the adjusted p-value was 0.05. Mean expression levels per sample group are stated in transcripts per million (TPM), calculated during quasi-mapping with the tool salmon. The list is sorted by gene expression.

gene name	L2FC	adjusted p-value	mean TPM empty virus con	mean TPM empty virus LPS	mean TPM APEX1(1-20) con	mean TPM APEX1(1-20) LPS
RGS5	-0,291	4,54E-02	1386,54	1138,17	1251,47	1178,35
MMRN1	-0,492	2,43E-02	888,40	637,40	859,18	699,02
VAPA	-0,205	3,21E-03	313,94	271,48	297,11	303,08
CKS2	-0,336	8,89E-03	287,78	228,74	288,97	258,79
PTTG1	-0,362	3,05E-02	285,06	221,31	276,16	228,63
NUCKS1	-0,368	3,27E-04	284,56	220,50	261,59	233,58
CNN3	-0,234	1,04E-02	273,16	231,87	274,05	257,51
CAPN2	-0,289	2,95E-06	260,46	212,68	252,84	228,05
PDCD6IP	-0,363	9,81E-04	189,22	147,31	181,29	183,72
IGF2BP3	-0,313	3,19E-02	182,15	146,90	163,66	171,08
ARL6IP1	-0,280	1,03E-02	162,45	133,86	160,85	137,37
SNX6	-0,454	3,97E-02	159,65	118,82	151,27	147,95
MCFD2	-0,277	7,05E-04	157,06	129,31	155,90	143,20
IPO5	-0,156	2,63E-02	149,62	133,94	146,54	145,32
LAMP2	-0,141	3,91E-02	147,87	133,68	145,02	147,45
PTK2	-0,273	4,98E-02	145,61	121,03	138,06	135,72
ARHGAP29	-0,385	4,42E-02	141,45	109,71	132,43	135,02
HP1BP3	-0,314	1,81E-02	139,47	112,32	131,88	120,54
RGS4	-0,258	5,00E-02	114,80	95,98	117,02	111,04
PRRC2B	-0,252	3,23E-02	112,97	94,70	107,19	104,11
CKAP5	-0,220	1,40E-02	107,89	92,55	102,33	94,75
VPS26A	-0,298	4,96E-02	107,19	87,14	98,37	102,76
TM7SF3	-0,245	1,94E-02	106,30	89,77	104,10	90,06
AHNAK	-0,388	1,41E-02	101,72	77,57	93,38	81,88
THOC3	-0,528	2,78E-02	101,44	70,86	81,58	81,55
NUDT4B	-0,160	4,73E-02	92,42	82,58	90,47	93,70
FAM114A1	-0,168	4,83E-02	92,21	81,82	91,47	90,41
ELK3	-0,267	3,36E-02	91,79	76,37	93,11	78,44
FLI1	-0,212	3,98E-02	90,93	78,68	90,90	82,08
MAPK1	-0,294	1,72E-02	88,64	72,59	86,09	85,87
SULT1B1	-0,498	7,96E-03	86,27	62,34	85,35	79,53
RAB14	-0,247	2,01E-03	85,29	71,61	83,80	80,96
DLGAP5	-0,621	7,41E-06	84,10	55,05	72,73	62,97
KPNA1	-0,182	4,83E-02	80,12	70,56	80,77	79,17
TMPO	-0,320	1,16E-03	79,02	63,21	73,14	75,58
AGFG1	-0,166	3,60E-02	78,64	69,89	77,52	78,78
PRR11	-0,326	5,24E-05	74,01	58,87	71,40	62,27
ARHGAP17	-0,465	2,43E-02	71,96	53,09	76,26	67,74
PTP4A1	-0,335	2,09E-02	71,32	56,77	71,78	69,82
AKT3	-0,276	3,23E-02	71,04	58,58	66,66	64,85
STEAP1B	-0,254	3,71E-02	70,66	59,27	73,25	67,94
FCF1	-0,213	3,43E-02	69,65	59,97	67,01	66,79
PGM2	-0,220	4,75E-02	67,65	58,03	64,30	59,35
NSD2	-0,239	1,18E-02	67,24	56,91	65,10	62,85
TOP2A	-0,435	2,01E-03	67,10	49,88	58,96	54,26

GLCE	-0,244	3,37E-02	66,72	56,47	67,09	61,35
EMCN	-0,524	2,95E-03	65,39	45,79	65,19	55,42
STEAP1	-0,346	7,86E-03	65,27	51,70	64,46	60,41
CD109	-0,318	4,92E-02	63,96	51,58	63,33	60,06
PRKAR2B	-0,307	4,99E-02	62,84	50,99	58,45	55,49
MYO5A	-0,276	8,91E-03	61,34	50,76	56,95	55,68
MSMO1	-0,536	6,33E-03	61,34	42,78	64,09	49,27
CDKN3	-0,614	2,21E-07	60,40	40,00	55,86	45,06
DOCK4	-0,288	3,91E-02	56,86	46,75	51,87	53,14
NPAS2	-0,270	4,45E-02	56,84	47,48	55,26	51,55
ASAP1	-0,339	8,50E-04	56,74	45,02	56,96	51,04
CKAP2	-0,456	3,39E-04	55,75	40,66	49,92	48,53
SPTLC1P1	-0,705	1,91E-03	54,20	33,52	41,77	47,38
UBA6	-0,346	3,56E-02	52,07	41,00	44,69	50,22
HNRNPLL	-0,211	2,37E-02	51,91	44,76	50,54	49,68
TM4SF18	-0,366	1,12E-02	51,79	40,24	51,66	46,59
PIGK	-0,369	8,75E-04	51,64	40,01	46,42	47,87
NDE1	-0,219	3,10E-02	49,68	42,66	48,99	43,31
CCNA2	-0,400	5,75E-03	48,48	36,81	47,63	40,45
MYH10	-0,217	9,83E-03	48,08	41,28	50,00	46,94
WDFY3	-0,381	6,18E-03	47,37	36,63	43,32	43,83
ARHGAP11A	-0,412	1,18E-02	46,93	35,56	40,52	42,96
R3HDM1	-0,468	1,00E-03	46,32	33,74	44,19	42,73
KNSTRN	-0,257	2,04E-02	45,96	38,50	43,46	40,62
CEP55	-0,538	2,21E-04	45,55	31,76	40,39	35,62
PBK	-0,449	1,23E-02	45,20	33,75	41,98	40,13
ARL5A	-0,701	2,40E-03	42,77	27,15	36,22	42,71
SQLE	-0,304	4,39E-02	42,63	34,49	42,79	37,29
CCNYL1	-0,395	5,43E-03	42,60	32,58	42,60	38,88
DHCR7	-0,348	3,00E-02	42,48	33,78	41,52	38,78
NRG1	-0,397	8,48E-03	42,39	31,95	35,24	34,48
NUSAP1	-0,257	4,10E-02	41,51	34,85	39,71	36,20
GLRX	-0,240	5,05E-03	41,14	34,71	39,91	36,15
NUF2	-0,646	2,35E-03	39,75	25,91	35,09	30,36
INSIG1	-0,278	5,37E-03	37,34	30,79	38,85	33,24
DARS2	-0,281	4,92E-02	37,20	30,55	34,90	34,55
SMAD5	-0,472	1,88E-02	36,84	26,75	32,33	33,09
ALDH3A2	-0,186	4,27E-02	36,02	31,66	37,27	34,20
EFCAB14	-0,243	7,20E-03	35,82	30,23	34,90	34,58
LPCAT2	-0,362	5,05E-03	35,25	27,55	35,88	32,97
SULT1E1	-0,482	1,43E-02	34,24	24,97	35,22	29,34
WASHC5	-0,200	3,11E-02	34,07	29,64	32,50	32,76
HMMR	-0,747	7,92E-04	33,34	20,52	27,10	24,57
MKI67	-0,525	2,87E-05	33,20	23,00	30,42	26,99
RACGAP1	-0,327	2,97E-02	32,94	26,26	30,63	28,43
POGLUT3	-0,247	4,29E-02	32,88	27,71	32,13	32,03
HIBCH	-0,436	4,24E-03	32,79	24,29	29,19	28,38
CENPF	-0,557	7,76E-04	32,77	22,82	24,86	24,63
PPP3CA	-0,232	3,15E-02	32,64	27,74	30,29	30,65
PHACTR4	-0,233	2,36E-02	31,22	26,47	30,90	27,69
NCEH1	-0,174	3,69E-02	31,00	27,51	29,64	29,31
SAMHD1	-0,369	3,92E-02	30,58	23,65	26,32	30,88
ACER3	-0,213	9,46E-03	28,85	24,84	27,65	28,00

YAP1	-0,249	4,42E-02	28,09	23,68	27,82	24,13
RBL2	-0,287	1,74E-02	28,00	22,97	27,75	25,92
MEF2A	-0,344	4,52E-02	25,86	20,49	26,06	24,22
DIAPH3	-0,353	2,53E-03	25,40	19,87	24,43	23,94
QTRT2	-0,279	6,99E-03	23,60	19,39	23,54	22,84
MLIP	-0,301	1,12E-02	23,51	19,05	21,92	19,99
MTMR10	-0,375	4,79E-04	23,37	17,98	25,88	21,54
INCENP	-0,443	6,18E-04	23,10	17,10	21,45	22,00
MPHOSPH9	-0,464	6,44E-03	23,10	16,83	19,80	20,44
SUV39H2	-0,517	1,93E-02	23,08	16,34	20,77	22,76
DEPDC1	-0,581	3,66E-02	22,60	15,59	20,95	18,12
FEM1B	-0,299	3,20E-02	22,23	18,09	20,12	20,75
CENPA	-0,407	1,53E-03	22,03	16,54	20,40	17,89
NEK2	-0,489	1,14E-03	22,02	15,84	19,40	16,29
ASPM	-0,784	1,93E-03	21,79	12,83	18,01	15,52
HMGCR	-0,321	3,30E-03	21,56	17,27	21,49	18,02
AFF1	-0,327	5,05E-03	21,49	17,22	20,68	20,16
SPAG5	-0,278	1,44E-02	21,35	17,53	20,53	18,15
VPS8	-0,319	3,23E-02	21,08	16,88	19,50	18,19
GPSM2	-0,652	2,34E-08	20,91	13,27	19,09	15,31
NECTIN3	-0,470	4,92E-03	20,80	15,17	19,88	18,09
RMND5A	-0,187	3,74E-02	20,62	18,11	19,45	20,02
WASF3	-0,313	1,93E-06	20,17	16,22	20,19	17,88
FNBP1	-0,269	1,05E-02	19,95	16,55	20,06	18,57
AC087721.2	-0,375	1,11E-03	19,71	15,18	18,38	16,98
RNMT	-0,425	6,33E-03	18,73	14,07	17,75	17,81
DAW1	-0,338	1,90E-02	18,59	14,68	18,49	15,57
APPBP2	-0,365	3,76E-02	18,50	14,48	17,97	17,14
LATS2	-0,211	1,24E-02	18,14	15,67	17,89	16,95
HSPE1-MOB4	-0,483	1,91E-03	18,08	12,83	14,01	17,11
TNRC18	-0,235	3,23E-02	17,96	15,27	16,98	16,51
COBLL1	-0,381	1,95E-03	17,94	13,75	18,11	18,74
KIF4A	-0,318	6,88E-03	17,68	14,14	16,63	14,60
ANGEL2	-0,371	2,33E-02	16,97	13,21	16,56	15,22
MEF2C	-0,347	5,59E-03	16,44	12,92	14,48	14,42
CEP70	-0,520	1,39E-02	16,00	11,25	14,09	12,74
GAS2L3	-0,436	2,59E-02	15,94	11,94	14,95	13,15
C2CD2	-0,279	2,28E-02	15,93	13,14	16,95	14,32
PIMREG	-0,446	9,83E-03	15,83	11,56	15,40	12,08
KNL1	-0,713	6,93E-04	15,79	9,70	13,00	12,47
ZNF800	-0,353	1,45E-02	15,76	12,34	15,31	14,62
LZTFL1	-0,296	4,42E-02	15,70	12,77	15,16	14,56
WASF1	-0,307	1,77E-02	14,78	11,94	15,34	14,30
PARPBP	-0,421	4,42E-02	14,56	11,09	13,45	13,05
SGO2	-0,656	1,49E-02	14,22	9,35	12,05	10,84
DEPDC1B	-0,341	1,19E-03	13,96	11,04	13,50	12,07
SENP1	-0,270	2,11E-02	13,76	11,38	12,95	13,53
RBBP9	-0,332	8,48E-03	13,73	10,93	14,11	13,07
COMMD3-BMI1	-0,606	3,22E-02	13,41	8,90	10,52	14,74
DIS3L	-0,276	3,98E-02	13,08	10,84	12,61	11,80
OIP5	-0,352	8,05E-03	12,74	9,96	12,37	10,39
CRYBG1	-0,333	8,42E-06	12,65	10,01	11,88	11,12
MGME1	-0,250	1,42E-03	12,53	10,52	12,16	11,54

ZCCHC2	-0,507	1,42E-04	12,17	8,65	11,66	9,51
SAMD8	-0,225	4,66E-02	12,12	10,39	12,15	11,68
CENPE	-0,750	3,78E-03	12,09	7,32	8,56	8,59
APAF1	-0,294	3,35E-03	10,95	8,93	10,43	10,60
SCLT1	-0,365	4,88E-02	10,30	8,06	9,18	8,74
SGO1	-0,365	2,94E-02	10,21	7,94	9,42	8,92
SMG1P3	-0,283	1,90E-02	9,83	8,09	9,21	8,92
BORA	-0,605	1,48E-03	9,52	6,33	8,54	7,95
GPD1L	-0,209	4,48E-02	9,38	8,12	9,50	8,38
SMG1P1	-0,304	4,00E-02	9,06	7,37	8,81	8,74
AC241640.1	-1,539	1,62E-02	8,80	3,10	10,71	4,34
ZNF791	-0,512	3,90E-04	8,68	6,12	8,47	8,46
PDE3A	-0,239	2,67E-02	8,68	7,37	8,38	7,77
HIVEP1	-0,366	1,74E-02	8,05	6,25	7,82	7,46
PLAC8	-0,413	2,19E-02	7,95	5,97	7,64	5,83
DPP4	-0,347	2,35E-03	7,75	6,08	7,84	6,65
CCDC190	-0,264	4,92E-02	7,46	6,19	7,29	6,82
IL1R1	-0,282	4,65E-02	7,25	6,00	7,21	7,81
KIF14	-0,564	6,99E-03	6,75	4,64	5,68	5,25
CXADR	-0,411	5,93E-04	6,35	4,78	5,87	5,17
MAP3K5	-0,280	3,78E-02	6,08	5,00	5,82	5,62
SYNJ1	-0,312	2,94E-03	6,07	4,89	5,73	5,43
TMSB15A	-0,521	1,77E-02	5,77	4,02	5,31	4,62
SFRP1	-0,311	1,62E-02	5,72	4,60	5,47	5,14
CDK19	-0,377	2,47E-02	5,68	4,36	5,76	4,81
ZBTB21	-0,333	4,44E-02	5,66	4,49	5,06	4,70
AP000295.1	-0,791	4,42E-02	5,60	3,52	5,37	5,08
MAP2K6	-0,373	1,13E-04	5,41	4,17	4,89	4,17
SPIN2B	-0,244	4,45E-02	5,22	4,40	5,07	4,63
RAB11FIP2	-0,544	9,61E-03	5,03	3,47	4,19	4,91
PIF1	-0,318	4,39E-02	5,01	4,02	4,92	4,13
MCM9	-0,297	2,33E-02	5,00	4,07	4,95	4,26
OCLN	-0,336	2,46E-02	4,99	3,94	5,14	4,58
RSC1A1	-0,388	3,96E-02	4,95	3,84	4,36	4,90
PIK3CG	-0,918	2,74E-02	4,87	2,59	3,73	3,36
KAT2B	-0,353	4,42E-02	4,34	3,43	4,45	3,95
ARNTL	-0,285	2,61E-02	4,25	3,48	3,91	3,68
ARHGAP11B	-0,429	1,57E-02	3,88	2,91	3,64	3,51
ELMOD1	-0,476	6,64E-04	3,50	2,52	3,34	2,71
ABCA8	-0,677	2,53E-03	3,31	2,07	3,08	2,19
MMP28	-1,256	3,11E-02	2,82	1,09	1,94	2,33
MYO5C	-0,322	2,37E-02	2,76	2,20	2,83	2,46
AL109918.1	-0,486	4,80E-04	2,58	1,85	2,46	2,03
UBN2	-0,561	1,44E-02	2,39	1,62	1,92	1,96
AC079594.2	-0,631	1,73E-02	2,29	1,48	1,89	2,29
SESN3	-0,641	1,72E-02	1,87	1,23	1,74	1,40
SMIM10	-0,426	4,12E-02	1,87	1,39	1,72	1,62
NBPF10	-0,326	7,85E-03	1,84	1,46	1,61	1,56
AC087632.2	-1,660	3,23E-04	1,79	0,53	1,83	1,71
CFAP69	-0,585	1,86E-02	1,66	1,12	1,54	1,45
RMDN2	-0,550	1,13E-02	1,65	1,14	1,48	1,52
H2AC6	-0,697	6,98E-03	1,58	0,98	1,56	1,14
SELP	-0,676	3,81E-02	1,48	0,93	1,36	0,99

CLDN10	-0,387	3,36E-02	1,16	0,89	1,25	0,96
SH3TC2	-0,555	2,35E-02	0,92	0,62	0,84	0,67
AMOT	-0,550	2,22E-02	0,72	0,49	0,64	0,53
ADAMTS12	-0,546	2,74E-02	0,71	0,49	0,67	0,62
CPA4	-0,847	1,94E-02	0,66	0,37	0,50	0,41
KCNJ15	-0,746	1,39E-03	0,61	0,36	0,34	0,37
WNK3	-0,654	1,77E-02	0,60	0,38	0,54	0,48
ADAM32	-1,239	8,91E-03	0,58	0,23	0,47	0,32
LPAR1	-0,631	2,38E-02	0,52	0,34	0,55	0,37
AC139530.2	-30,000	3,86E-10	0,41	0,00	0,28	0,21
H2AC11	-0,650	1,32E-02	0,34	0,22	0,33	0,22
SLC26A4	-0,719	3,05E-02	0,34	0,21	0,33	0,22
SCUBE3	-0,411	2,69E-02	0,25	0,19	0,26	0,20
GP1BB	-22,691	1,38E-06	0,23	0,00	0,34	0,21
SPTA1	-1,154	2,10E-02	0,17	0,08	0,11	0,10
FBXO15	-1,172	2,43E-02	0,16	0,07	0,14	0,11
CMKLR1	-1,002	4,91E-02	0,13	0,07	0,10	0,07
DNM1P47	-0,477	2,40E-02	0,12	0,09	0,10	0,10

Supplementary Table S9: Genes upregulated by LPS exclusively in cells that express APEX1(1-20). To identify genes upregulated by LPS specifically in cells expressing moderate levels of APEX1(1-20), but not in cells transduced with the empty virus, the results of the DGE analysis of both cells populations after treatment with detoxified (control) or active LPS (LPS) were com-bined. The L2FC (Log 2-fold change) states the average difference in gene expression between both treatments, positive values denote upregulation by LPS treatment. Wald test from DESeq2 was used to calculate the significance of the change in the expression. The adjusted p-values take the number of tested genes into account, the threshold for the adjusted p-value was 0.05. Mean expression levels per sample group are stated in transcripts per million (TPM), calculated during quasi-mapping with the tool salmon. The list is sorted by gene expression.

gene name	L2FC	adjusted p-value	mean TPM empty virus con	mean TPM empty virus LPS	mean TPM APEX1(1-20) con	mean TPM APEX1(1-20) LPS
IL1RL1	0,254	4,90E-03	186,63	200,07	175,35	209,27
SELENOT	0,254	3,59E-03	139,92	144,17	139,26	165,99
PXDN	0,668	1,34E-08	130,73	158,35	107,72	171,11
YIPF5	0,269	4,19E-03	85,82	92,90	86,53	104,21
DPYSL3	0,257	2,43E-02	88,43	94,98	83,51	99,79
NPTN	0,193	6,51E-03	67,85	72,68	67,70	77,38
COLGALT1	0,367	8,71E-03	55,94	68,32	56,83	73,29
PLSCR1	0,208	3,55E-02	49,76	53,82	50,49	58,32
ABI3BP	0,365	1,22E-02	42,83	48,38	48,73	62,49
CLEC1A	0,182	4,06E-02	49,73	54,56	48,64	55,20
CD44	0,503	5,30E-03	54,57	62,30	47,62	68,26
TUSC3	0,294	6,19E-03	41,13	44,21	39,24	48,10
ANGPT2	0,361	8,20E-05	38,50	43,80	34,41	44,26
DUXAP9	0,284	2,13E-02	18,14	19,50	18,29	22,28
NUAK1	0,442	3,93E-02	21,46	20,17	17,49	23,93
QPCT	0,564	1,72E-03	19,62	20,56	16,30	24,34
ATAD2	0,366	2,64E-02	14,35	15,01	13,40	17,11
IFI44	0,499	1,71E-02	12,54	15,99	12,14	17,09
HPSE	0,245	6,15E-03	10,00	11,08	9,86	11,67
PRIM1	0,506	6,36E-04	12,13	12,56	9,75	13,82
AK4	0,170	2,29E-02	9,80	10,28	9,68	10,88
CHAF1A	0,466	2,83E-02	9,93	11,66	9,48	13,05
NID2	0,901	9,23E-06	12,01	15,22	8,98	16,73
LRP12	0,252	2,81E-02	8,67	9,31	8,70	10,36
RGS2	0,398	4,63E-02	9,44	10,39	8,45	11,13
ARSK	0,211	4,04E-02	8,45	8,43	8,09	9,35
GK	0,285	2,52E-02	7,60	8,71	7,35	8,95
ZFPM2	0,369	3,39E-02	7,57	8,13	6,70	8,72
IL7R	0,419	2,96E-04	6,01	6,97	5,92	7,92
CLSPN	0,378	3,55E-02	5,33	6,04	5,66	7,33
EXO1	0,511	1,98E-03	5,92	6,37	5,28	7,53
CENPBD1	0,199	3,14E-02	4,71	4,52	4,22	4,85
CREB5	0,326	5,89E-04	2,77	3,01	2,82	3,52
PDE1C	0,280	4,63E-02	2,55	3,03	2,57	3,12
DNAH11	0,366	3,50E-02	2,47	2,47	2,19	2,81
AUNIP	0,412	4,00E-02	1,93	2,39	1,88	2,50
ALDH1L2	0,311	1,70E-02	2,04	2,30	1,85	2,29
LY75-CD302	0,547	1,38E-02	2,12	1,73	1,78	2,57
CACHD1	0,277	2,91E-02	1,81	2,00	1,76	2,13
SH3BP1	0,715	6,40E-03	1,32	1,89	1,40	2,31
CHRNA1	0,426	1,79E-02	1,13	1,44	1,26	1,69
TUBB2B	0,445	4,84E-02	1,12	1,35	1,08	1,47
TCF7	0,391	1,78E-02	1,07	1,19	1,05	1,38
NLRC3	0,376	1,24E-03	0,86	1,03	0,83	1,08
ARHGEF26	0,351	4,67E-02	0,63	0,64	0,58	0,74

RASGRF2	0,347	3,88E-02	0,57	0,71	0,56	0,71
SLC6A15	0,655	1,04E-02	0,37	0,53	0,40	0,64
AMPD3	0,780	2,81E-02	0,38	0,39	0,37	0,66
SLIT3	0,552	1,16E-02	0,23	0,31	0,25	0,36
AFF3	1,033	9,24E-03	0,30	0,35	0,23	0,46
PRRT2	2,229	2,83E-02	0,18	0,23	0,17	1,00
MYPN	0,620	2,17E-02	0,11	0,15	0,09	0,14
OASL	1,044	1,05E-02	0,07	0,09	0,08	0,17
PAPPA2	1,562	1,69E-04	0,06	0,09	0,04	0,12
PLA2G5	1,414	2,80E-02	0,02	0,08	0,03	0,08
TMEM184A	1,991	6,69E-04	0,04	0,05	0,02	0,06
AC019117.4	8,471	1,17E-02	0,47	0,18	0,00	0,26
CR354443.1	32,453	1,50E-11	0,08	0,18	0,00	0,20
H3P6	30,910	1,34E-10	1,80	3,13	0,00	13,28
SRP9P1	25,646	5,29E-07	20,60	15,67	0,00	8,97
AC244260.1	8,622	1,48E-02	0,10	0,20	0,00	0,21

Supplementary Table S10: Genes downregulated by LPS exclusively in cells that express APEX1(1-20). To identify genes downregulated by LPS specifically in cells expressing moderate levels of APEX1(1-20), but not in cells transduced with the empty virus, the results of the DGE analysis of both cells populations after treatment with detoxified (con) or active LPS (LPS) were combined. The L2FC (Log 2-fold change) states the average difference in gene expression between both treatments, negative values denote downregulation by LPS treatment. Wald test from DESeq2 was used to calculate the significance of the change in the expression. The adjusted p-values take the number of tested genes into account, the threshold for the adjusted p-value was 0.05. Mean expression levels per sample group are stated in transcripts per million (TPM), calculated during quasi-mapping with the tool salmon. The list is sorted by gene expression.

gene name	L2FC	adjusted p-value	mean TPM empty virus con	mean TPM empty virus LPS	mean TPM APEX1(1-20) con	mean TPM APEX1(1-20) LPS
NQO1	-0,281	1,26E-02	511,43	442,40	520,05	427,72
PIR	-0,566	2,25E-02	239,69	198,51	259,77	176,08
IGFBP4	-0,294	1,95E-04	187,16	172,64	197,36	160,86
CLEC14A	-0,190	1,99E-02	174,69	168,66	185,74	162,74
SOX18	-0,314	1,70E-05	54,38	50,61	57,60	46,31
TCN2	-0,295	4,29E-02	35,46	32,01	35,93	29,35
BTG2	-0,190	4,93E-02	30,69	28,95	32,58	28,57
CDC25B	-0,240	3,55E-02	28,01	24,68	28,15	23,83
ATP2B4	-0,175	2,48E-02	26,61	24,37	26,48	23,44
NOS3	-0,330	1,99E-02	25,85	21,96	25,64	20,42
GIMAP8	-0,227	3,68E-02	22,98	20,67	24,54	20,96
GGT5	-0,353	3,66E-03	22,00	20,89	24,12	18,92
SHE	-0,219	3,05E-02	22,25	20,13	22,92	19,69
ENOSF1	-0,301	2,71E-02	20,15	17,83	22,46	18,19
C1orf115	-0,180	3,90E-02	20,75	19,57	20,95	18,48
GJA4	-0,568	1,75E-04	14,65	12,07	19,24	12,98
KLF2	-0,494	4,28E-07	13,78	14,54	16,08	11,40
BTD	-0,185	4,29E-02	14,47	12,87	14,48	12,75
RAMP2	-0,465	4,90E-03	11,52	10,04	14,27	10,32
GIMAP1	-0,265	1,26E-02	11,67	11,04	12,66	10,53
BCAM	-0,275	1,74E-02	11,25	10,30	11,90	9,84
ABCG2	-0,578	1,24E-03	9,23	7,10	11,11	7,46
CYP1A1	-0,508	1,91E-02	10,46	8,05	10,60	7,45
IFT122	-0,270	2,29E-02	9,27	8,45	10,30	8,58
NFIA	-0,403	3,63E-02	7,98	7,49	9,43	7,14
MXD3	-0,387	4,04E-02	7,99	6,69	8,09	6,15
TM7SF2	-0,335	3,14E-02	6,80	6,27	6,85	5,42
ZNF467	-0,453	3,40E-03	5,68	4,91	6,20	4,54
HSPB6	-0,351	2,83E-02	4,44	4,25	5,03	3,95
MRAP2	-0,384	4,81E-03	3,70	3,13	4,15	3,17
ZNF219	-0,385	2,52E-02	3,65	2,93	3,90	2,98
SPACA6	-0,405	9,24E-03	3,56	3,10	3,69	2,80
LRTOMT	-0,402	5,51E-03	2,33	2,41	3,68	2,78
ANKRD44	-0,767	3,45E-02	2,23	2,28	3,61	2,12
GPX3	-0,573	3,61E-03	2,36	2,07	3,17	2,13
ASS1	-0,446	4,39E-03	2,43	2,14	2,98	2,19
GSTM2	-0,342	2,92E-02	2,59	2,20	2,86	2,26
MAP3K14	-0,384	1,70E-02	2,62	2,14	2,78	2,13
NRROS	-0,303	9,64E-04	2,48	2,40	2,65	2,15
STC1	-0,462	2,58E-02	2,79	2,23	2,33	1,69
FAM221A	-0,310	3,55E-02	2,04	1,88	2,23	1,80
CRACR2B	-0,419	1,65E-04	1,73	1,72	1,98	1,48
PARD6A	-0,362	3,74E-02	1,79	1,69	1,86	1,44
SLCO2A1	-0,674	2,74E-02	1,48	1,32	1,82	1,15
LRRC75A	-0,393	1,30E-03	1,45	1,25	1,58	1,20

GUCY1A1	-0,911	1,65E-02	1,09	0,83	1,55	0,84
SMIM3	-0,424	6,42E-03	1,27	1,14	1,40	1,05
NTSR1	-0,347	3,46E-02	1,41	1,20	1,33	1,05
C20orf204	-0,498	1,04E-02	1,08	0,97	1,17	0,83
TCF15	-0,467	7,58E-03	1,05	0,97	1,13	0,82
LHX6	-0,471	1,15E-02	1,04	0,83	1,05	0,76
FCF1P2	-23,428	3,30E-11	1,19	1,42	1,05	0,00
AP005018.2	-23,882	3,43E-06	0,91	1,50	1,03	0,00
CCL23	-0,826	3,88E-02	0,99	0,74	1,02	0,57
COL1A2	-0,415	1,87E-03	0,77	0,64	1,01	0,76
CASKIN1	-2,314	1,30E-02	0,36	0,10	0,91	0,29
WNT9A	-0,439	2,83E-02	0,74	0,63	0,85	0,63
MATN2	-0,529	4,63E-02	0,58	0,43	0,76	0,53
DLL1	-0,370	2,69E-02	0,63	0,58	0,70	0,54
C4B	-1,377	7,99E-03	0,51	0,53	0,67	0,25
CD36	-0,760	2,93E-02	0,49	0,31	0,58	0,34
EIF3CL	-0,720	9,24E-03	0,43	0,30	0,54	0,32
FBLN2	-0,677	1,05E-02	0,44	0,32	0,51	0,32
ABCC2	-0,663	1,63E-02	0,38	0,36	0,50	0,32
CA4	-1,172	1,37E-02	0,28	0,19	0,41	0,19
EGLN3	-1,171	1,62E-03	0,31	0,25	0,38	0,17
ARL14EPL	-0,995	4,63E-02	0,29	0,23	0,38	0,19
CDH4	-0,476	1,48E-02	0,33	0,26	0,37	0,26
INHBB	-0,814	1,69E-04	0,22	0,18	0,30	0,17
CLEC3B	-1,195	2,74E-02	0,17	0,11	0,28	0,12
AC005520.3	-1,051	5,63E-03	0,25	0,19	0,26	0,14
KCNN4	-0,792	3,04E-02	0,20	0,17	0,24	0,14
ABLIM2	-1,741	7,58E-03	0,17	0,13	0,24	0,07
CAMSAP3	-0,935	1,63E-02	0,07	0,08	0,17	0,09
SYT7	-0,953	3,29E-02	0,15	0,10	0,16	0,08
TBC1D3K	-30,254	2,79E-10	0,06	0,08	0,08	0,00
RASSF10	-1,470	2,80E-02	0,03	0,02	0,06	0,02
SCNN1B	-2,117	1,78E-02	0,04	0,03	0,04	0,01
ADAMTS15	-1,383	1,02E-03	0,03	0,02	0,04	0,02
NOS1	-2,260	2,64E-02	0,00	0,00	0,02	0,00
RTN4RL1	-2,401	3,55E-02	0,01	0,01	0,01	0,00
